# Acupuncture for Relieving Abdominal Pain and Distension in Acute Pancreatitis: A Systematic Review and Meta-Analysis

**DOI:** 10.3389/fpsyt.2021.786401

**Published:** 2021-12-03

**Authors:** Fengya Zhu, Shao Yin, Xinyun Zhu, Deya Che, Zimeng Li, Yue Zhong, Hui Yan, Daohui Gan, Lanying Yang, Xiaohan Wu, Liuying Li

**Affiliations:** ^1^Traditional Chinese Medicine Department, Zigong First People's Hospital, Zigong, China; ^2^Clinical Medical School, Hospital of Chengdu University of Traditional Chinese Medicine, Chengdu, China; ^3^Traditional Chinese Medicine Department, The People's Hospital of Leshan, Leshan, China; ^4^Acupuncture and Tuina School, The Third Teaching Hospital, Chengdu University of Traditional Chinese Medicine, Chengdu, China

**Keywords:** acupuncture, acute pancreatitis, abdominal pain, abdominal distension, systematic review, meta-analysis

## Abstract

**Background:** Clinical evidence suggests that acupuncture is effective for relieving abdominal pain and distension in acute pancreatitis (AP). However, there is a lack of systematic reviews and meta-analyses that provide high-quality evidence of the efficacy and safety of acupuncture in this context.

**Aim:** To assess the efficacy and safety of acupuncture for relieving abdominal pain and distension in AP.

**Methods:** We searched the PubMed, Web of Science, Embase, Cochrane Library, CNKI, Wanfang, VIP, and China Biomedical Literature databases. Randomized controlled trials of acupuncture plus routine treatment (RT) vs. RT alone or RT plus sham/placebo acupuncture were included. Primary outcomes included total effectiveness rate, VAS scores for abdominal pain and distension, and time until relief of abdominal pain and distension. Secondary outcomes included time until recovery of bowel sound, time until first defecation, length of hospital stay, and APACHE II score.

**Results:** Nineteen eligible original studies (*n* = 1,503) were included. The results showed that acupuncture in combination with RT had a significant advantage in terms of increasing the total effectiveness rate [risk ratio: 1.15; 95% confidence interval (CI): 1.06–1.24; *P* = 0.001]. Acupuncture also reduced the VAS score for abdominal pain [weighted mean difference (WMD): −1.45; 95% CI: −1.71 to −1.19; *P* < 0.0001] and the VAS score for abdominal distension (WMD: −0.71; 95% CI: −1.04 to −0.37; *P* < 0.0001) in patients with AP. Other results also showed the efficacy of acupuncture. One study reported adverse events after acupuncture.

**Conclusion:** Acupuncture in combination with RT has a better effect than RT alone for relieving abdominal pain and distension in AP. More rigorous studies are needed to confirm this result.

**Systematic Review Registration:** PROSPERO CRD42019147503 (https://www.crd.york.ac.uk/PROSPERO/display_record.php?RecordID=147503).

## Introduction

Acute pancreatitis (AP) is one of the commonest acute abdominal conditions and is the leading cause of hospitalization for gastrointestinal disorders in the United States and many other countries. Gallstones and alcohol abuse are longstanding risk factors for AP (1). Although the mortality rate of AP has declined over the past decade, the hospitalization rate has steadily increased (2). It has been reported that the average global incidence of AP is 34/100,000 [95% confidence interval (CI): 23–49/100,000] with no statistically significant difference between men and women. The incidence of AP varies worldwide. North America and the Western Pacific (as defined by the WHO) are the regions where AP is commonest (3).

AP often involves gastrointestinal dysfunction, which may cause systemic inflammatory response syndrome or even multiple organ dysfunction syndrome (4, 5). Abdominal pain and distension are the main clinical symptoms of AP, and the symptoms of patients with severe AP (SAP) are more serious (6). Therefore, improving gastrointestinal function is important for recovery from AP. At present, opioids are the main treatment that is used for pain control in patients with AP. However, there is a lack of clear practical guidelines for the use of opioids, and drug dependence easily occurs (7). I In the United States in 2018 drug overdoses caused 67,367 deaths, of which 46,802 (69.5%) were due to opioid overdoses (8). It is therefore urgently necessary to find alternative therapies for alleviating gastrointestinal dysfunction in patients with AP.

Acupuncture is an important form of traditional Chinese medicine (TCM). Studies have shown that acupuncture can improve gastrointestinal function, enhance gastric motility, and effectively treat abdominal pain (9, 10). The use of acupuncture to treat AP is recommended by the Consensus on Acute Pancreatitis Management of Chinese Medicine (11). In 2019 a systematic review (SR) and meta-analysis (MA) investigated the efficacy and safety of acupuncture in the treatment of AP but did not focus on the relief of abdominal pain and distension in patients with AP and only included a few studies that reported abdominal pain and distension (12). Some randomized controlled trials (RCTs) have provided clinical evidence that acupuncture relieves abdominal pain and distension in AP. However, there is a lack of relevant SRs that provide high-quality evidence for clinical decision-making. Therefore, this study aimed to examine the efficacy and safety of acupuncture for relieving abdominal pain and distension in AP.

## Materials and Methods

### Types of Study Selected

This study included RCTs of acupuncture for relieving abdominal pain and distension in patients with AP regardless of whether blinding or allocation concealment was used. There were no restrictions on language. Case reports, reviews, animal studies, clinical research studies that directly compared different kinds of acupuncture, and studies of combination treatment involving therapies other than acupuncture were excluded. The protocol of the SR and MA was registered before the study was conducted (CRD42019147503: https://www.crd.york.ac.uk/PROSPERO/display_record.php?RecordID=147503). The study was conducted according to the guidelines of the Preferred Reporting Items for Systematic Reviews and Meta-Analyses Statement (13). The authors have completed the PRISMA reporting checklist (Supplementary Material 3).

### Types of Patient Included

Patients diagnosed with AP in accordance with internationally accepted diagnostic criteria for AP (both mild and severe types) were included. All participants needed to have been hospitalized for acupuncture treatment. Subjects who were under 18 years of age and pregnant patients were excluded. No other conditions were imposed.

### Types of Intervention Included

Acupuncture was a supplementary treatment provided in addition to routine treatment (RT). Traditional acupuncture, electroacupuncture, warm acupuncture, ear acupuncture, and moxibustion were included. The details of the acupuncture method were clearly explained in accordance with the Standards for Reporting Interventions in Clinical Trials of Acupuncture, including the selection of the needle, acupoints, manipulations, and course of treatment (14). The control intervention comprised RT alone or RT plus sham/placebo acupuncture. The control group received the same RT as the acupuncture group.

### Types of Outcome Measure

The outcome indicators reported in the original study included the results of the use of acupuncture for relieving abdominal pain and distension in AP.

The primary outcomes included the total effectiveness rate, visual analog scale (VAS) scores for abdominal pain and distension, and time until relief of abdominal pain and distension. The secondary outcomes included the time until recovery of bowel sound, time until first defecation, length of hospital stay, Acute Physiology and Chronic Health Evaluation II (APACHE II) score, and adverse events.

### Search Strategy

This study searched eight databases, namely, the PubMed, Web of Science, Embase, Cochrane Library, China National Knowledge Infrastructure, Wanfang, VIP, and SinoMed databases. We selected eligible studies that had been published as of July 31, 2021. The search terms that were used were acute pancreatitis, abdominal pain, abdominal distension, acupuncture, electroacupuncture, moxibustion, ear acupuncture, warm acupuncture, and randomized controlled trial. The search strategy was adjusted according to the characteristics of each database (Supplementary Material 1). In addition, we manually searched the references in all relevant original articles to identify other qualifying studies. Gray literature was difficult to obtain. All eligible studies were evaluated by experts in the relevant fields and were finally analyzed.

### Selection of Studies

Two reviewers (LYY and XHW) screened the original studies according to the retrieval strategy and imported them into EndNote X9 (Clarivate Analytics, Philadelphia, PA, USA) to exclude duplicate studies. Subsequently, non-qualifying studies were excluded on the basis of the title and abstract. Finally, according to the inclusion criteria and after the full text had been read, eligible RCTs were selected for further evaluation (DHG and HY). All researchers worked independently. Any disputes were settled between the two researchers, and any unresolved differences were resolved by a third reviewer (LLY).

### Data Extraction and Quality Assessment

After the study selection had been finalized, two reviewers (ZML and YZ) independently extracted data that included the publication year, lead author, language, funding, trial registration, ethical review, sample size, age, and gender, course of disease, interventions, acupoints, duration of treatment, and primary and secondary outcomes. Another reviewer (FYZ) tried to contact the study authors to acquire data that were necessary for the MA. All data were cross-checked after extraction, and any disagreements were resolved by the third reviewer (LLY). We used the approach according to the Grading of Recommendations Assessment, Development, and Evaluation (GRADE) system to assess the quality of evidence of each result (SY and XYZ). The quality of evidence was divided into four levels, namely, high, moderate, low, and very low (15).

### Assessment of Risk of Bias

Two researchers (FYZ and SY) individually assessed the risk of bias using the Cochrane risk of bias tool (16). The assessment included: (1) generation of random sequences; (2) allocation concealment; (3) blinding of participants and research personnel; (4) blinding to outcome assessment; (5) incomplete outcomes data; (6) selective reporting; and (7) other forms of bias. The risk of bias in each domain was graded as high, low, or unclear on the basis of relevant information extracted from each eligible study. Any disagreements were resolved by discussion, and any unresolved disagreements were settled by the third researcher (LLY).

### Statistical Analysis

Stata 15.0 software was used for statistical analysis. The risk ratio (RR) and the corresponding 95% CI were used for dichotomous variables. Continuous data were presented as the weighted mean difference (WMD) or standardized mean difference (SMD) with the corresponding 95% CI values. The *I*^2^ statistic and *P*-value were used to determine whether heterogeneity was present among the results. If *I*^2^ > 50% or *P* < 0.05, heterogeneity was significant, and hence a random-effects model was adopted (17). Otherwise, a fixed-effects model was adopted. If significant heterogeneity was present, subgroup analysis or meta-regression was performed to discover the source of the heterogeneity. Moreover, sensitivity analysis was used to test the stability of the results (18). Finally, publication bias was assessed using a funnel plot and Egger's-test (19). If significant publication bias was found, the stability of the results was tested using the trim-and-fill method (20).

## Results

### Trial Characteristics

**Figure 1:**
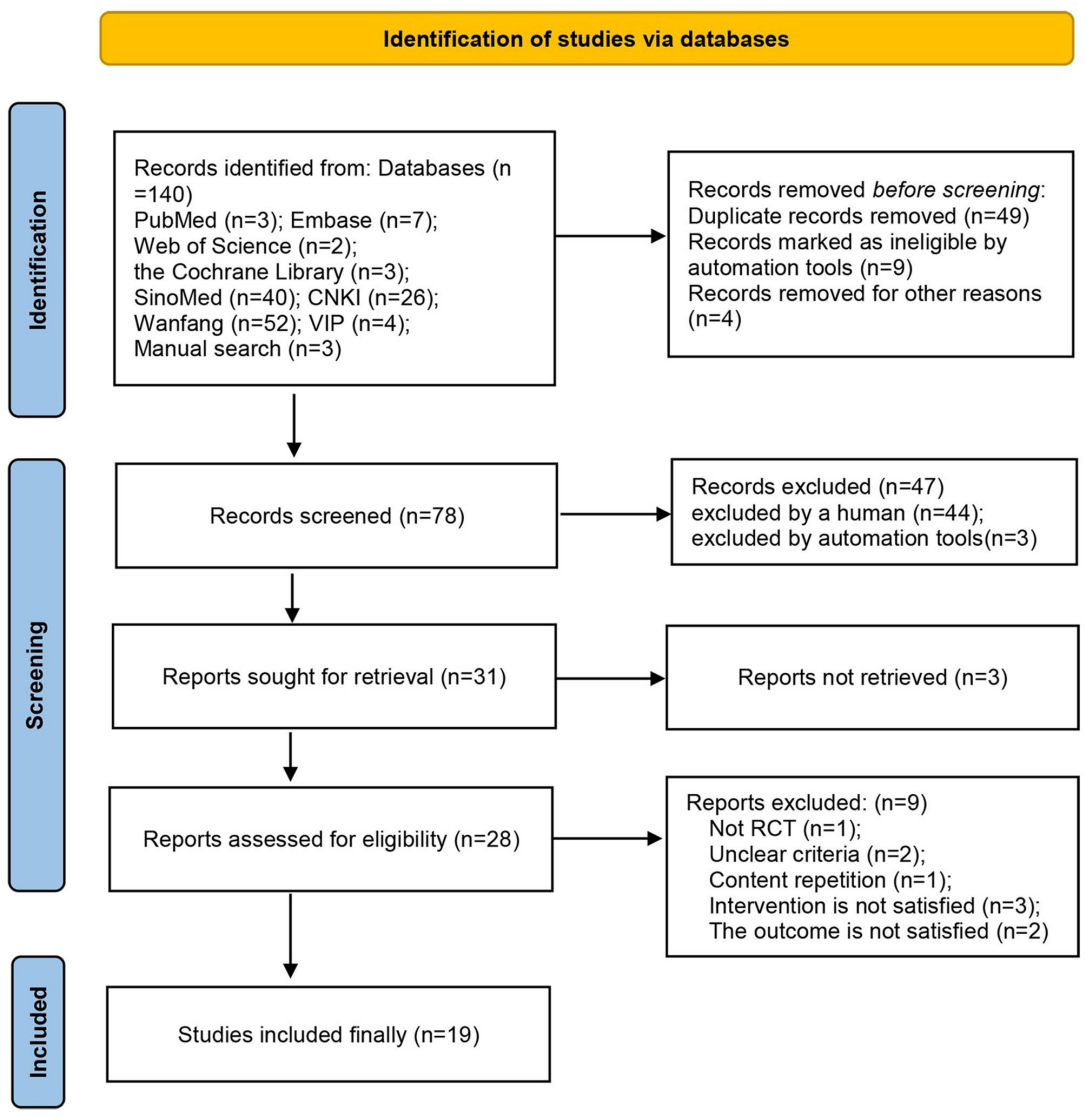
Flow chart of the study.

According to the search strategy, 133 studies that were retrieved from the eight abovementioned databases were initially screened. Nineteen RCTs (21–39) with a total of 1,503 participants were finally included in this study (Figure 1). All these studies were published between 2009 and 2021 and were carried out in China. The sample sizes ranged from 38 to 232, and the duration of treatment was 3–14 days. The control groups received RT, whereas the treatment groups received acupuncture in combination with RT. In nine RCTs (21, 23, 29, 30, 35–39) patients were treated with acupuncture; in seven (22, 24–26, 28, 32, 34) patients were treated with electroacupuncture; and in three RCTs (27, 31, 33) patients were treated with moxibustion. Adverse events were mentioned in five studies (24, 28, 32, 35, 37) (Table 1). The exclusion list is shown in Supplementary Material 2.

**Table 1:** Characteristics of the included trials.

**References**	**Sample size (E/C)**	**Age [y, mean (SD)] (E/C)**	**Sex (male/female) (E, C)**	**Experimental treatment**	**Control treatment**	**Acupuncture points**	**Duration, d**	**Key outcome**
Xu and Liao (21)	112/120	Not reported	Not reported	Acupuncture + TCM + RT	TCM + RT	Zusanli (ST36) Neiguan (PC6) Zhongwan (RN12) Tianshu (ST25) Pishu (BL20) Weishu (BL21)	5	④, ⑤, ⑥, ⑧
Luo et al. (22)	48/20	43.2/44.8	30/18, 13/7	EA + RT	RT	Zhongwan (RN12) Zusanli (ST36) Neiguan (PC6) Hegu (LI4) Quchi (LI11) Tianshu (ST25)	3–6	①, ④, ⑤, ⑥, ⑦, ⑧, ⑨
Su et al. (23)	26/29	Not reported	Not reported	Acupuncture + RT	RT	Zusanli (ST36)	3–5	④, ⑤, ⑦, ⑧
Sang et al. (24)	19/19	46.37 (8.28)/47.20 (11.06)	13/6, 11/8	EA + RT	RT	Zusanli (ST36) Taichong (LR3)	10	②, ③, ⑥, ⑦, ⑨
Zhang (25)	20/20	44.3 (16.2)/43.2 (17.4)	17:3/15:5	EA + RT	RT	Zusanli (ST36) Neiguan (PC6) Liangqiu (ST34) Zhongwan (RN12) Hegu (LI4)	7	④, ⑥, ⑦
Li (26)	40/40	42.7 (12.8)/43.4 (11.9)	25/15, 24/16	EA + RT	RT	Zusanli (ST36) Shangjuxu (ST37) Xiajuxu (ST39) Fenglong (ST40) Tianshu (ST25) Zhongwan (RN12) Zhigou (SJ6) Hegu (LI4) Taichong (LR3)	10	①, ⑤, ⑥, ⑧
Li (27)	35/35	Not reported	19/16, 21/14	Moxibustion + RT	RT	Zhongwan (RN12) Zusanli (ST36)	7	③, ⑦, ⑧
Li et al. (28)	70/70	46 (11)/47 (10)	43/27, 35/35	EA + RT	RT	Zusanli (ST36) Zhigou (SJ6)	5	②, ③
Liu et al. (29)	49/48	47.37 (12.82)/48.83 (12.92)	Not reported	Acupuncture + RT	RT	Hegu (LI4) Tianshu (ST25) Daheng (SP15) Guanyuan (RN4) Zusanli (ST36) Shangjuxu (ST37) Xiajuxu (ST39) Yanglingquan (GB34) Fenglong (ST40) Taichong (LR3)	10	④, ⑦, ⑧
Han and Wang (30)	21/21	48.92 (11.74)/48.95 (11.81)	13/8, 13/8	Acupuncture + RT	RT	Tianshu (ST25) Dachangshu (BL25) Shangjuxu (ST37) Quchi (LI11) Hegu (LI4) Zhongwan (RN12) Zusanli (ST36) Neiguan (PC6) Zhigou (SJ6)	3	④, ⑥, ⑦
Chen (31)	30/30	Not reported	Not reported	Moxibustion + RT	RT	Shenque (RN8) Zusanli (ST36) Neiguan (PC6)	7	④, ⑥, ⑦
Luo (32)	30/30	49.63 (7.31)/49.9 (6.15)	13/17, 14/16	EA + RT	RT	Zusanli (ST36) Zhongwan (RN12)	5	①, ②, ③, ④, ⑤, ⑥, ⑦, ⑧, ⑨
Wang (33)	30/30	47.10 (12.78)/46.60 (11.60)	14/16, 12/18	Moxibustion + RT	RT	Zhongwan (RN12) Shenque (RN8) Zusanli (ST36)	7	④, ⑦, ⑧
Zhao et al. (34)	60/60	63 (8)/63 (8)	32/28, 36/24	EA + RT	RT	Dachangshu (BL25) Shangjuxu (ST37)	14	①, ④, ⑥, ⑦, ⑧, ⑨
Feng (35)	32/32	50.13 (14.98)/49.34 (12.135)	23/9, 21/11	Acupuncture + RT	RT	Danzhong (RN17) Zhongwan (RN12) Qihai (RN6) Xuehai (SP10) Zusanli (ST36) Waiguan (SJ5) Xingjian (LR2) Yinlingquan (SP9)	5	①, ②, ③
Zheng and Wu (36)	48/48	Not reported	Not reported	Acupuncture + RT	RT	Zusanli (ST36)	10	③, ⑦, ⑧
Chen (37)	33/33	45.39 (12.02)/44.00 (10.80)	21/1, 22/10	Acupuncture + RT	RT	Zusanli (ST36) Tianshu (ST25) Xiajuxu (ST39) Sanyinjiao (SP6) Qihai (RN6) Zhongwan (RN12)	5	③
Jia (38)	26/26	47.5 (18.129)/50.462 (17.716)	19/7, 12/14	Acupuncture + RT	RT	Zusanli (ST36) Hegu (LI4)	7	④, ⑥, ⑦, ⑨
Huang et al. (39)	31/32	45.55 (6.68)/46.56 (7.02)	14/17, 14/18	Acupuncture + RT	RT	Zusanli (ST36) Jinggu (BL64) Dazhong (KI4) Yinlingquan (SP9) Qichong (ST30)	5	①, ④, ⑤, ⑥, ⑦, ⑧

①*Total effectiveness rate; ②VAS score for abdominal pain; ③VAS score for abdominal distension; ④Time until relief of abdominal pain; ⑤Time until relief of abdominal distension; ⑥Time until recovery of bowel sound; ⑦Time until first defecation; ⑧Length of hospital stay; ⑨APACHE II score*.

*E, experimental group; C, control group; EA, electro acupuncture; RT, routine treatment; TCM, Traditional Chinese medicine*.

### Risk of Bias

**Figure 2:**
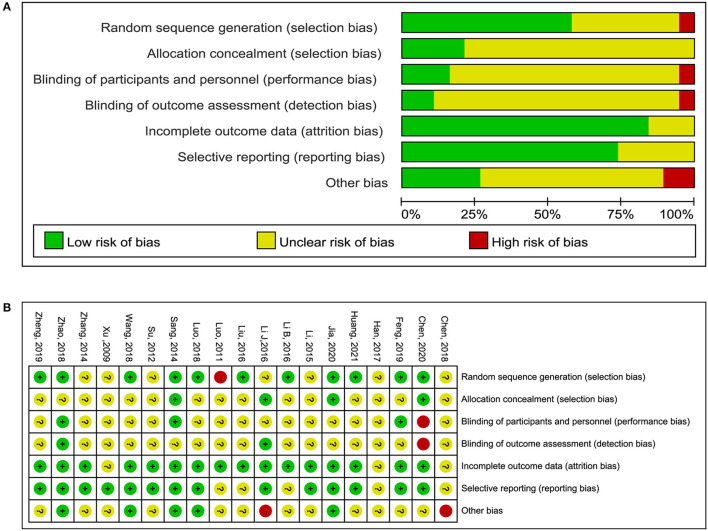
Risk of bias graph. **(A)** Risk of bias item presented as percentages across all included RCTs. **(B)** Risk of bias item for included RCTs.

All 19 RCTs were assessed for risk of bias (Figure 2). Eleven RCTs had a low risk of bias in the generation of random sequences; four RCTs had a low risk of bias in allocation concealment, whereas in the other RCTs the risk of bias in this domain was unclear; three RCTs reported blinding of participants and research personnel; two RCTs reported blinding to outcome assessment, of which one study was classified as having a high risk of bias, whereas in the other study the risk of bias was unclear; 16 RCTs had a low risk of bias in incomplete outcomes data; and 14 RCTs had a low risk of bias in selective reporting.

### Primary Outcomes

#### Total Effectiveness Rate

**Figure 3:**
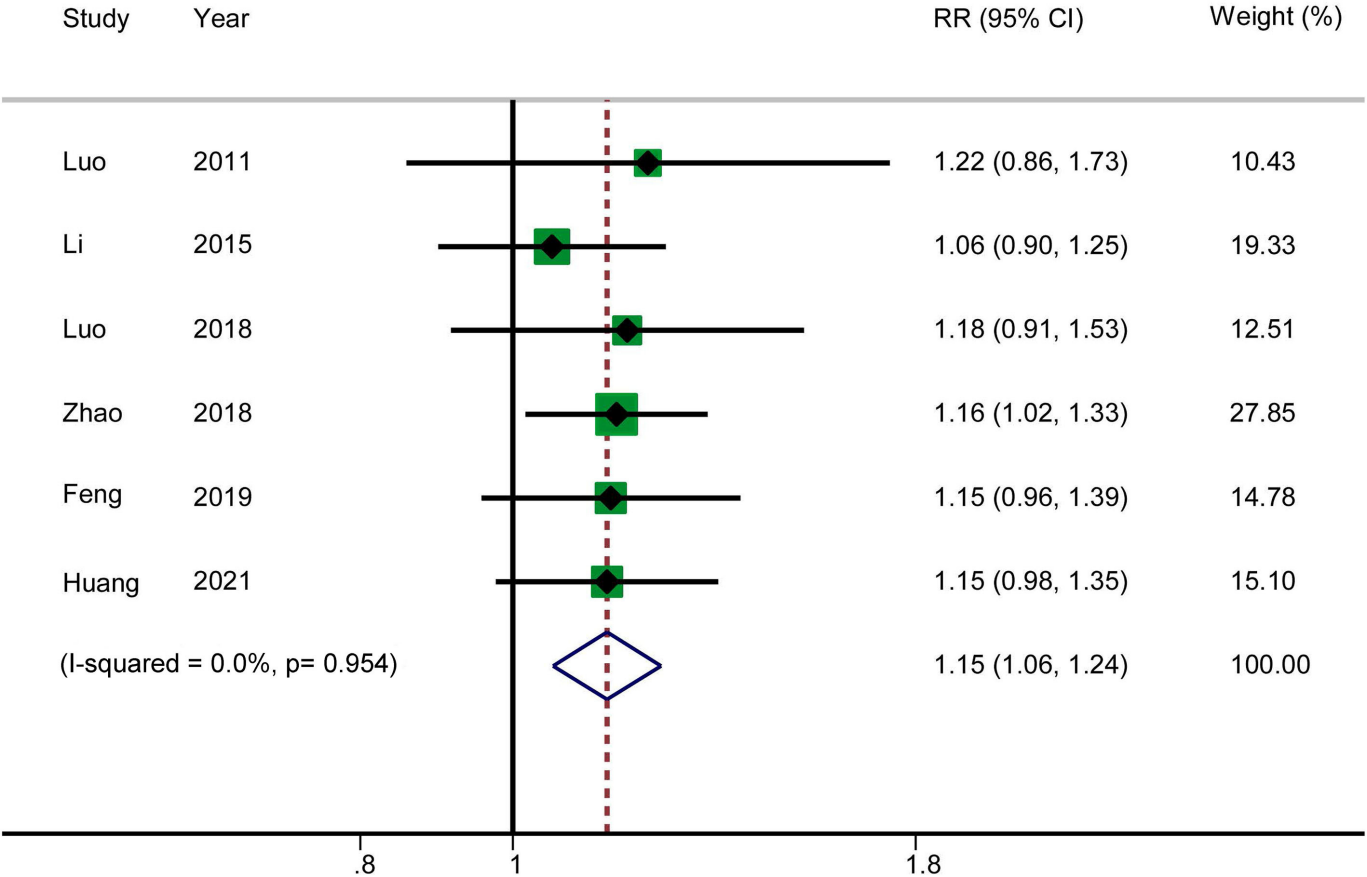
Meta-analysis of total effectiveness rate.

Six RCTs reported the total effectiveness rate. The results showed that acupuncture in combination with RT increased the total clinical effectiveness rate in patients with AP (RR: 1.15; 95% CI: 1.06–1.24; *P* = 0.001). Heterogeneity was not significant (*I*^2^ = 0.0%, *P* = 0.954), and hence a fixed-effects model was used to merge the data (Figure 3). The GRADE quality of this evidence was low.

#### VAS Score for Abdominal Pain

**Figure 4:**
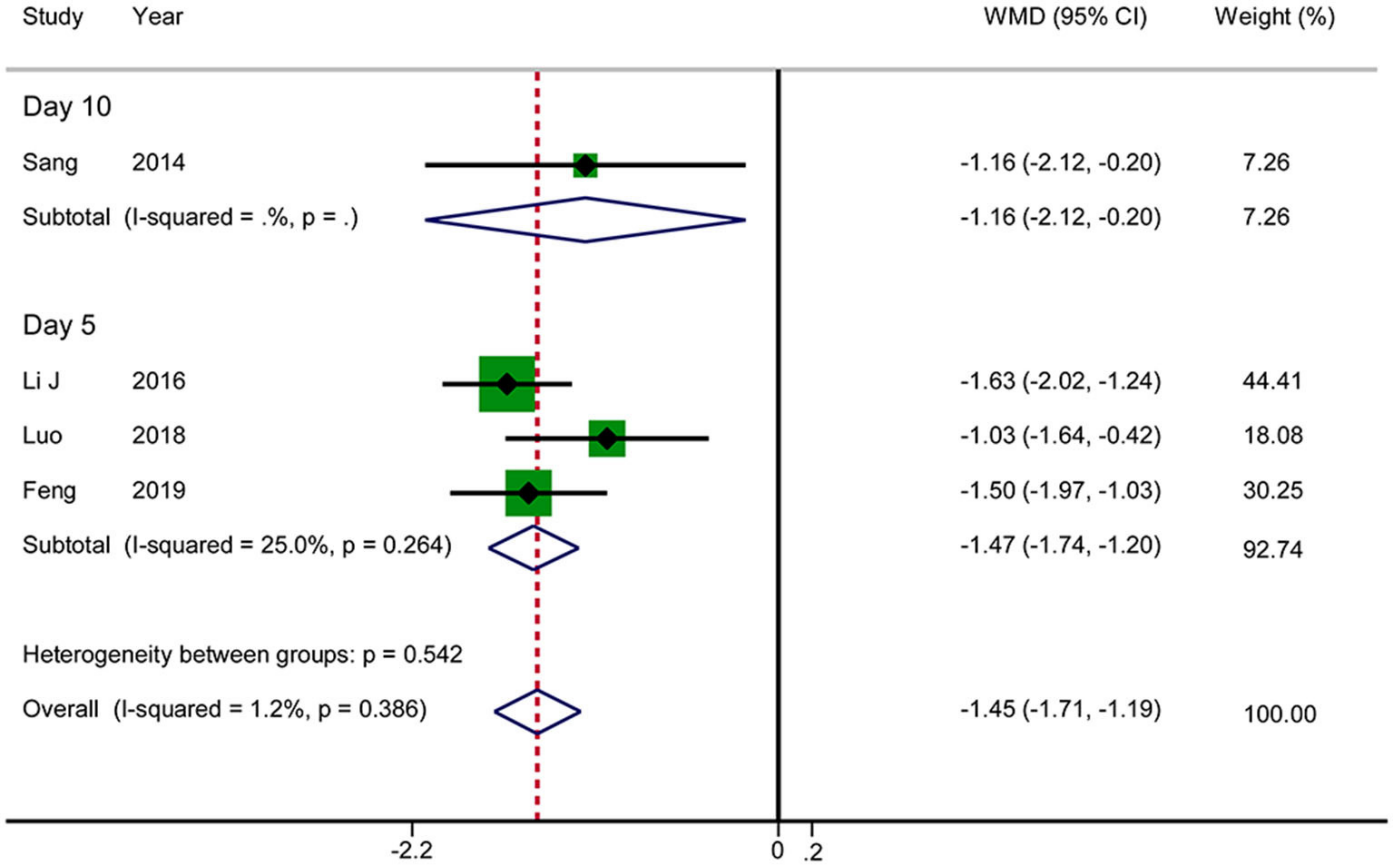
Meta-analysis of VAS score for abdominal pain.

Four RCTs reported the VAS score for abdominal pain. The results showed that acupuncture reduced the VAS score for abdominal pain in patients with AP (WMD: −1.45; 95% CI: −1.71 to −1.19; *P* < 0.0001). No significant heterogeneity was observed (*I*^2^ = 1.2%, *P* = 0.386), and hence the data were combined using a fixed-effects model. There was an inconsistency in the time at which the outcome indicators were measured between various studies (day 5 after treatment in three RCTs vs. day 10 after treatment in one RCT). We therefore performed a subgroup analysis, according to which acupuncture was associated with lower VAS scores in the day 5 group in comparison with RT (WMD: −1.47; 95% CI: −1.74 to −1.20; *P* < 0.0001) (Figure 4). The GRADE quality of this evidence was low.

#### VAS Score for Abdominal Distension

**Figure 5:**
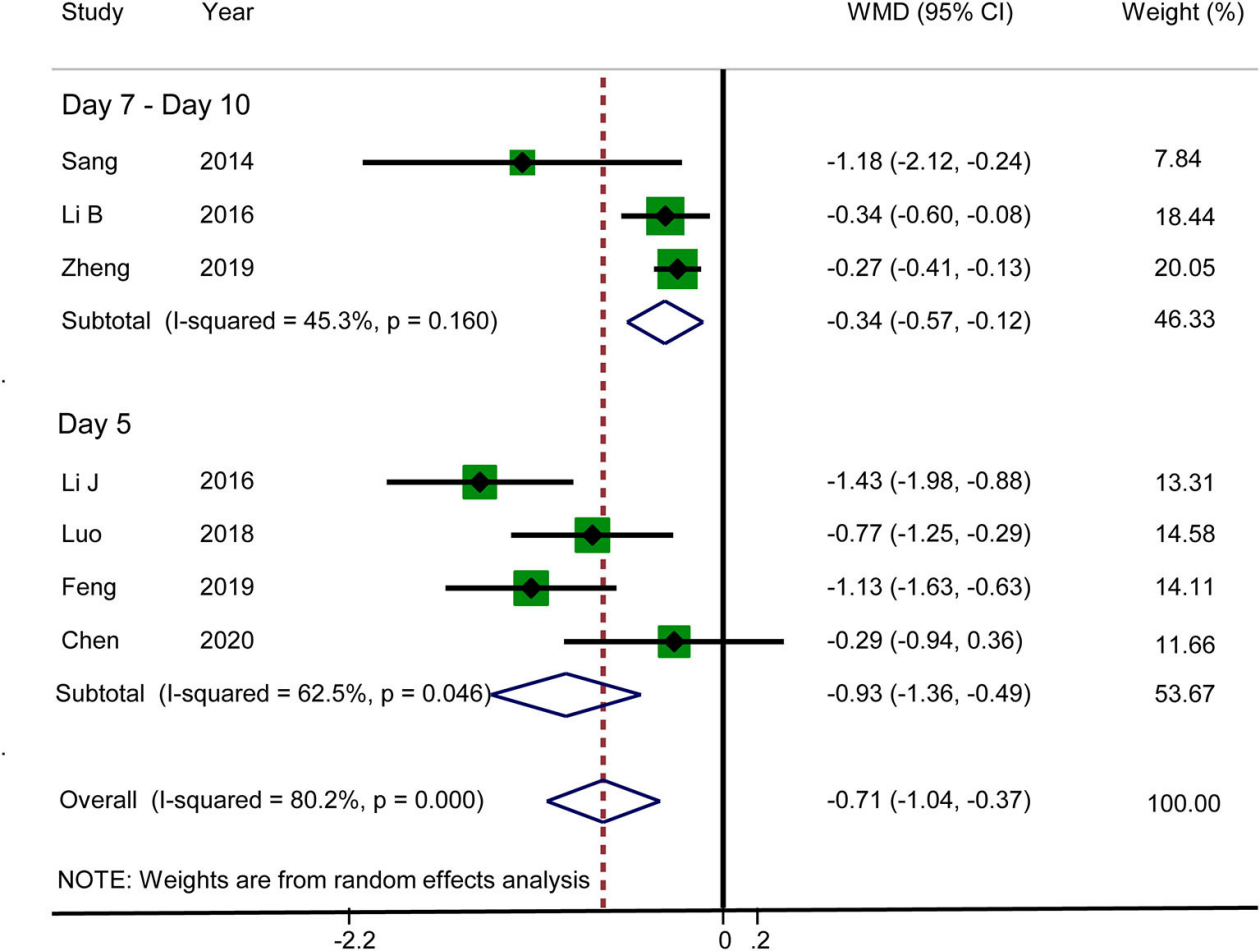
Meta-analysis of VAS score for abdominal distension.

Seven RCTs reported that acupuncture reduced the VAS score for abdominal distension (WMD: −0.71; 95% CI: −1.04 to −0.37; *P* < 0.0001). Heterogeneity was significant (*I*^2^ = 80.2%, *P* < 0.0001), and hence a random-effects model was adopted. Therefore, we conducted a subgroup analysis on the basis of different observation times. The data for subgroup 1 (day 5) and subgroup 2 (days 7–10) were WMD: −0.93; 95% CI: −1.36 to −0.49; *P* < 0.0001 and WMD: −0.34; 95% CI: −0.57 to −0.12; *P* < 0.0001, respectively, which suggested that the VAS score for abdominal distension was lower in the acupuncture group. There was no significant heterogeneity in subgroup 2 (*I*^2^ = 45.3%, *P* = 0.160), but there was significant heterogeneity in subgroup 1 (*I*^2^ = 62.5%, *P* = 0.046) (Figure 5). Sensitivity analysis was used to further investigate the sources of heterogeneity. After the successive exclusion of each study, none of the studies affected the pooled analysis. The GRADE quality of this evidence was very low.

#### Time Until Relief of Abdominal Pain

**Figure 6:**
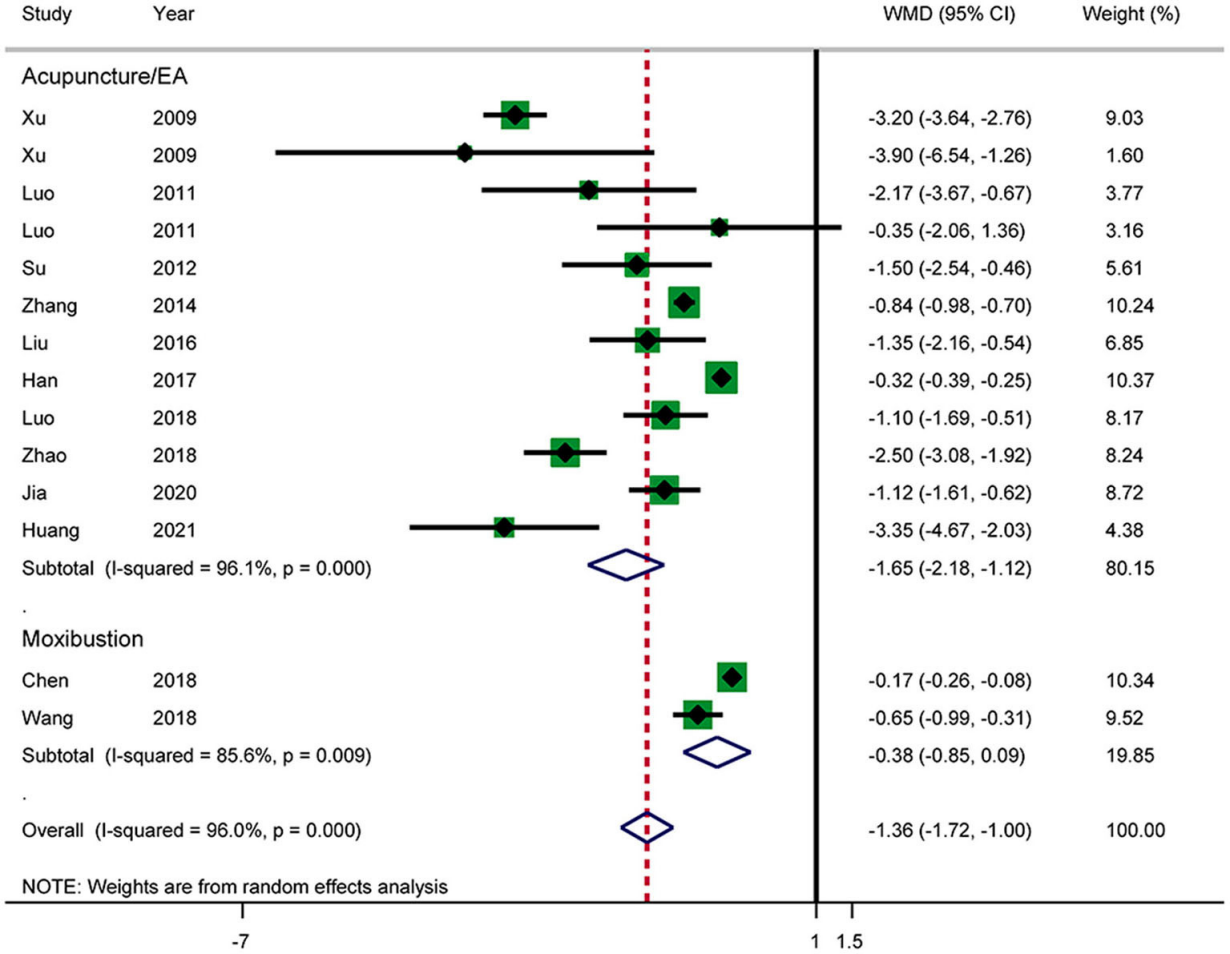
Meta-analysis of time until relief of abdominal pain.

Twelve RCTs reported the time until relief of abdominal pain (Figure 6). The results showed that acupuncture shortened the time until relief of abdominal pain in patients with AP (WMD: −1.36; 95% CI: −1.72 to −1.00; *P* < 0.0001). Heterogeneity was high (*I*^2^ = 96.0%, *P* < 0.0001), and a random-effects model was therefore used. Subgroup analysis by intervention showed a difference between the acupuncture and electroacupuncture groups (WMD: −1.65; 95% CI: −2.18 to −1.12; *P* < 0.0001). No such effect was observed in the moxibustion group (WMD: −0.38; 95% CI: −0.85 to −0.09; *P* = 0.110). Heterogeneity was still significant according to the subgroup analysis. No source of heterogeneity was found by sensitivity analysis. The GRADE quality of this evidence was very low.

#### Time Until Relief of Abdominal Distension

**Figure 7:**
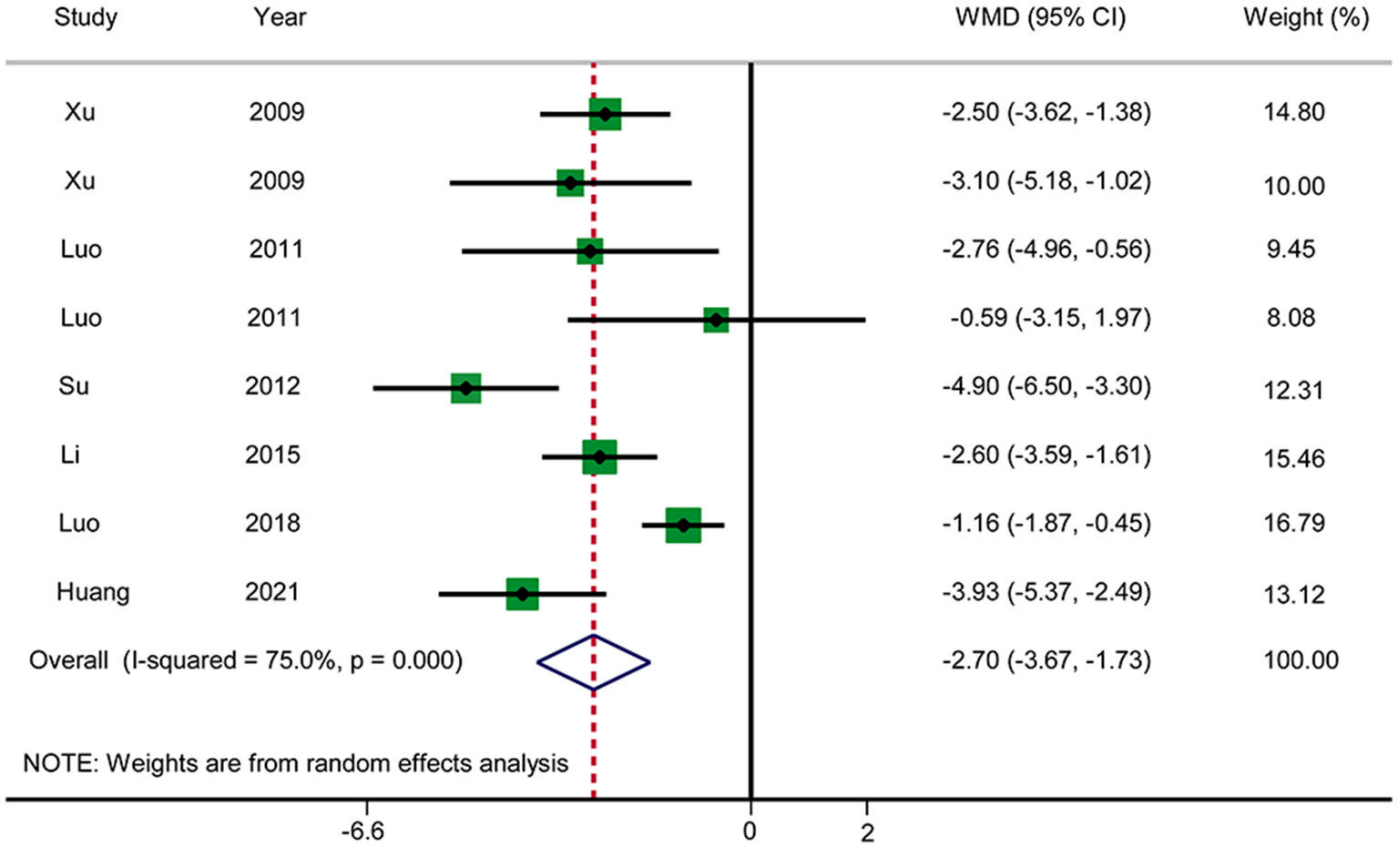
Meta-analysis of time until relief of abdominal distension.

Six RCTs reported the time until relief of abdominal distension (Figure 7). The pooled results showed that acupuncture reduced the time until relief of abdominal distension in patients with AP in comparison with RT (WMD: −2.70; 95% CI: −3.67 to −1.73; *P* < 0.0001). Heterogeneity was significant (*I*^2^ = 75.0%, *P* < 0.0001), and hence a random-effects model was used. We selected the duration of treatment and year of publication as covariates to perform meta-regression. No source of heterogeneity was found, and no abnormalities were found by sensitivity analysis. The GRADE quality of this evidence was very low.

### Secondary Outcomes

#### Time Until Recovery of Bowel Sound

**Figure 8:**
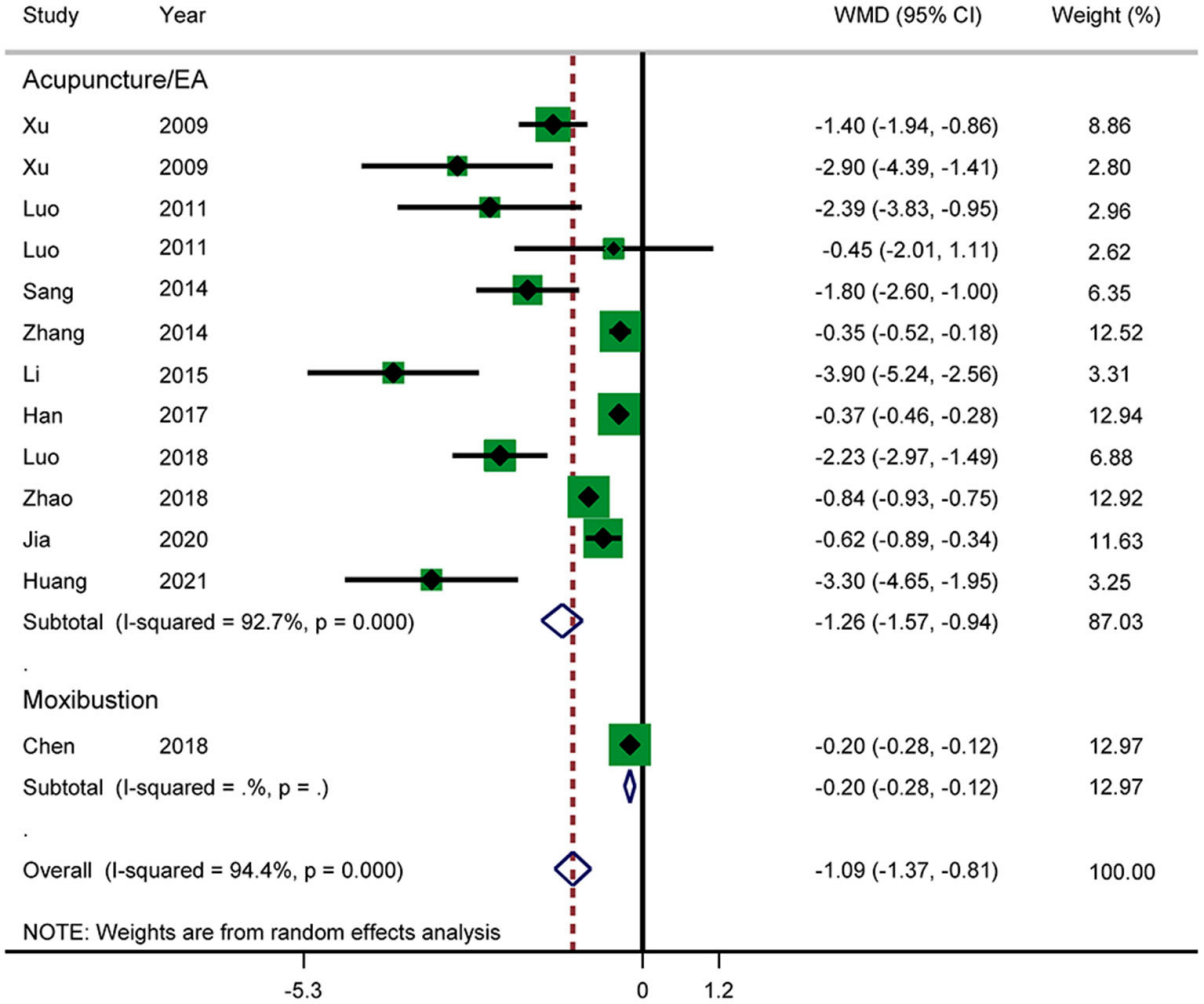
T Meta-analysis of time until recovery of bowel sound.

Eleven RCTs reported the time until recovery of bowel sound (Figure 8). The results showed that acupuncture in combination with RT shortened the time until recovery of bowel sound (WMD: −1.09; 95% CI: −1.37 to −0.81; *P* < 0.0001). Heterogeneity was significant (*I*^2^ = 94.4%, *P* < 0.0001), and a random-effects model was therefore used. According to a subgroup analysis by intervention, the results showed that there was a significant difference between the acupuncture and electroacupuncture groups (WMD: −1.26; 95% CI: −1.57 to −0.94; *P* < 0.0001). The subgroup analysis also suggested that there was significant heterogeneity in the results. No source of heterogeneity was found by sensitivity analysis. The GRADE quality of this evidence was very low.

#### Time Until First Defecation

**Figure 9:**
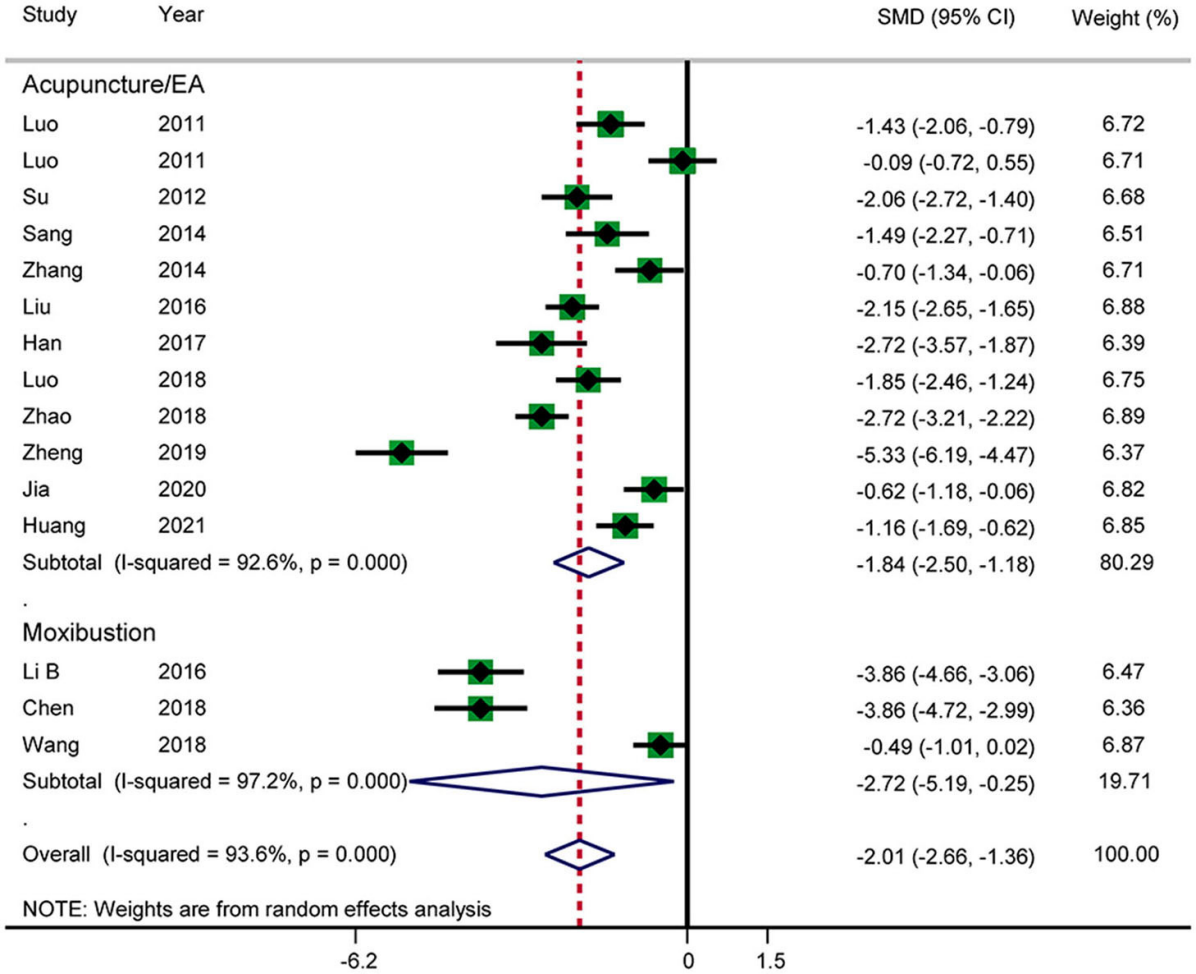
Meta-analysis of time until first defecation.

Fourteen RCTs reported the time until first defecation (Figure 9). The results showed that acupuncture shortened the time until first defecation (SMD: −2.01; 95% CI: −2.66 to −1.36; *P* < 0.0001). Heterogeneity was significant (*I*^2^ = 93.6%, *P* < 0.0001), and a random-effects model was therefore used. According to a subgroup analysis by intervention, there was a significant difference between the acupuncture and electroacupuncture groups (SMD: −1.84; 95% CI: −2.50 to −1.18; *P* < 0.0001) and the moxibustion group (SMD: −2.72; 95% CI: −5.19 to −0.25; *P* = 0.031), and heterogeneity was high. No source of heterogeneity was found by further sensitivity analysis. The GRADE quality of this evidence was very low.

#### Length of Hospital Stay

**Figure 10:**
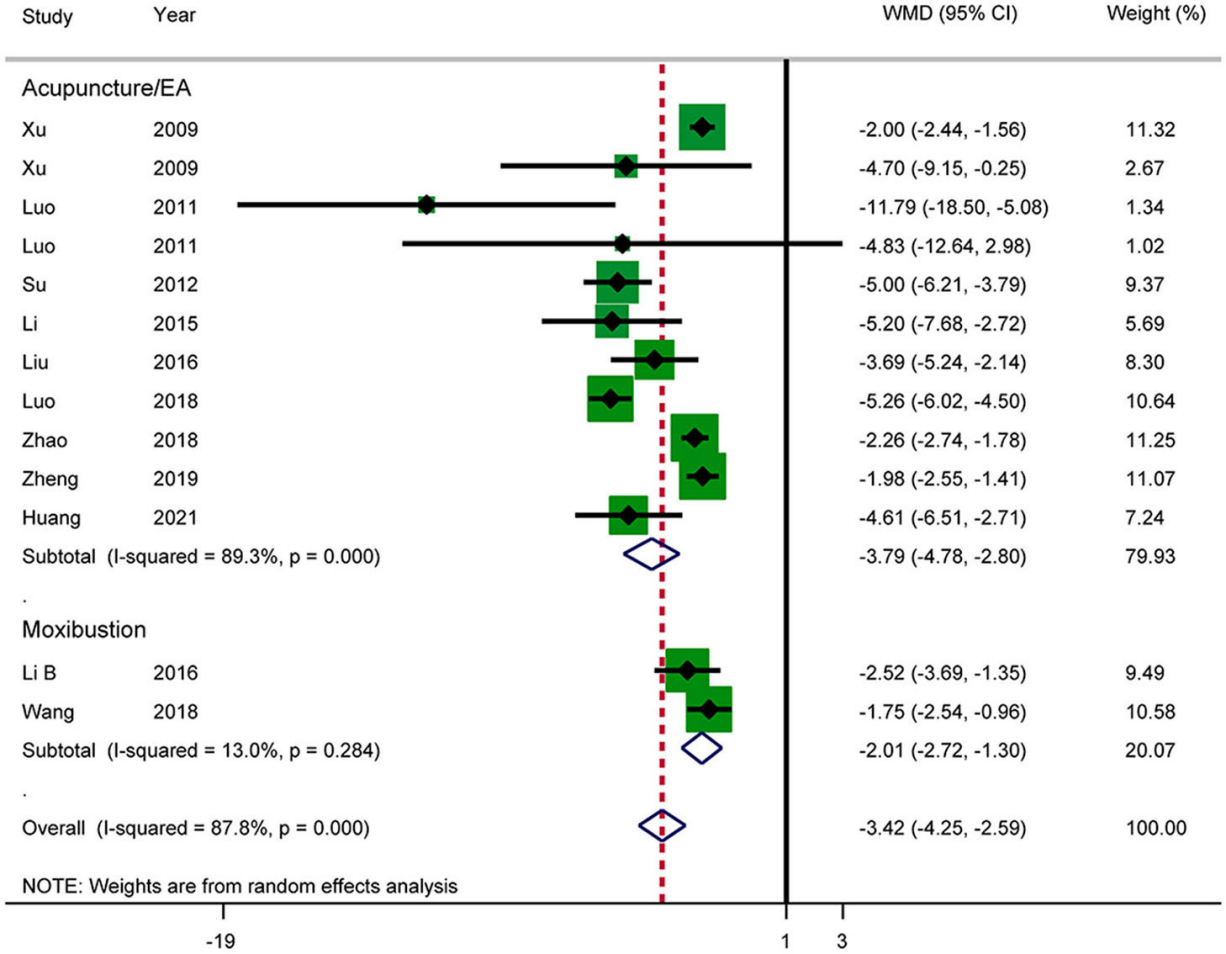
Meta-analysis of length of hospital stay.

Eleven RCTs reported the length of hospital stay (Figure 10). The results showed that acupuncture reduced the length of hospital stay in patients with AP (WMD: −3.42; 95% CI: −4.25 to −2.59; *P* < 0.0001). Heterogeneity was significant (*I*^2^ = 87.8%, *P* < 0.0001), and a random-effects model was therefore used. According to a subgroup analysis by intervention, there was a significant difference between the acupuncture and electroacupuncture groups (SMD: −3.79; 95% CI: −4.78 to −2.80; *P* < 0.0001) and the moxibustion group (SMD: −2.01; 95% CI: −2.72 to −1.30; *P* = 0.031). The heterogeneity in the moxibustion group was lower (*I*^2^ = 13.0%, *P* = 0.284). Further sensitivity analysis showed that the results were stable. The GRADE quality of this evidence was very low.

#### APACHE II Score

**Figure 11:**
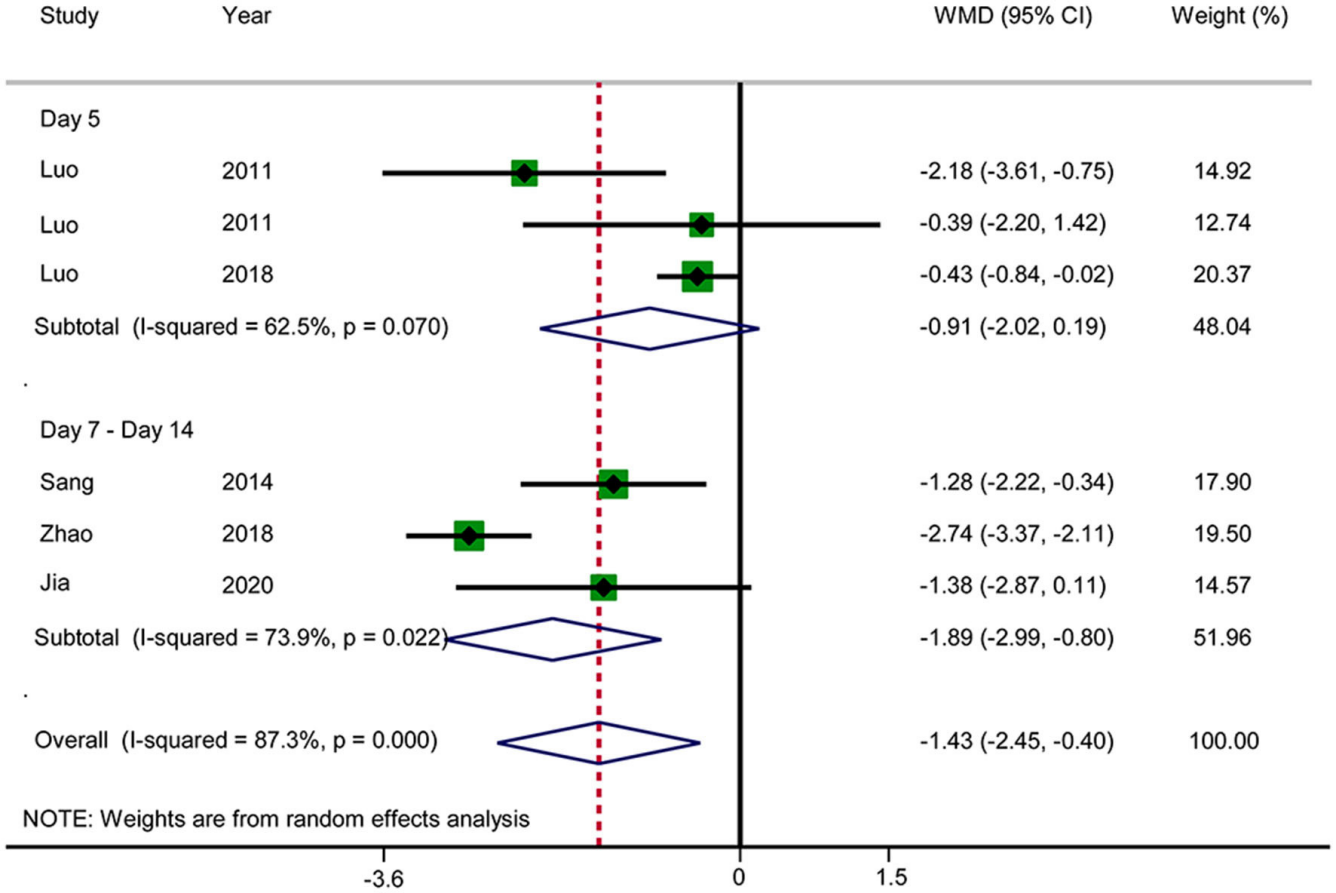
Meta-analysis of APACHE II score.

Six RCTs reported the APACHE II score (Figure 11). The results showed that acupuncture reduced the APACHE II score (WMD: −1.43; 95% CI: −2.45 to −0.40; *P* = 0.006). Heterogeneity was significant (*I*^2^ = 87.3%, *P* < 0.0001), and a random-effects model was therefore used. Subgroup analysis was conducted by observation time (days 5 vs. 7–14). No significant difference in the results was observed in the day 5 group (WMD: −0.91; 95% CI: −2.02 to −0.19; *P* = 0.106), but a significant difference was observed in the days 7–14 group (WMD: −1.89; 95% CI: −2.99 to −0.80; *P* = 0.001). Subgroup analysis did not substantially reduce the heterogeneity. No source of heterogeneity was found by sensitivity analysis. The GRADE quality of this evidence was very low.

### Risk of Publication Bias

**Figure 12:**
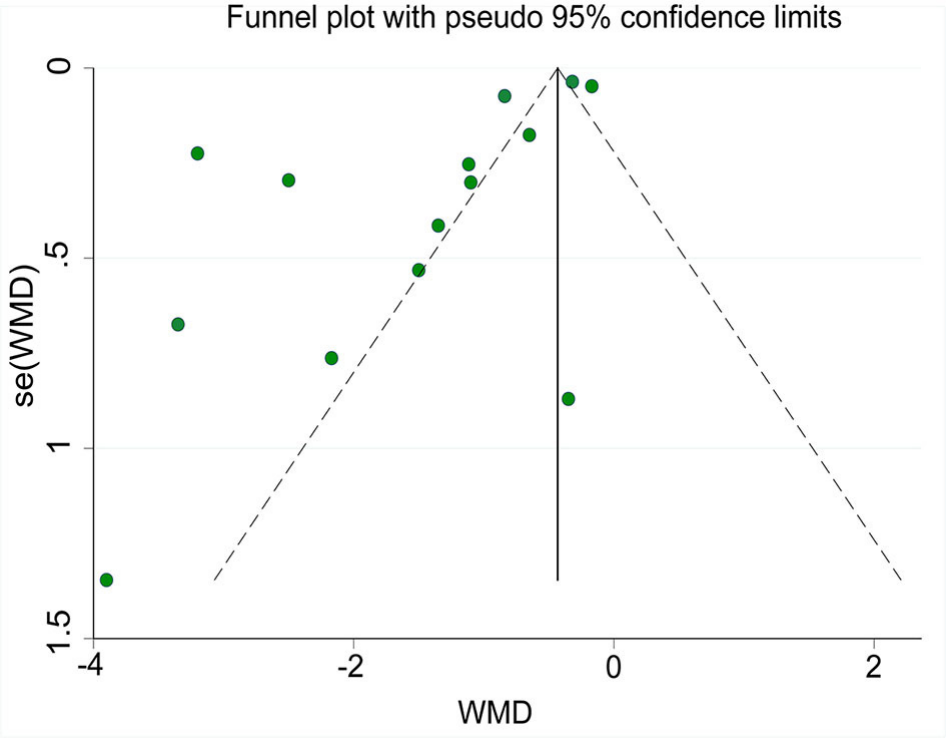
Funnel plot of the time until relief of abdominal pain.

A funnel plot of the time until relief of abdominal pain is shown in Figure 12. Egger's-test indicated that publication bias was present. Sensitivity analysis using the trim-and-fill method showed that the results were stable.

### Adverse Events

Of the 19 RCTs, five mentioned adverse events, of which four noted that no treatment-related adverse events occurred in the acupuncture group. Chen (37) noted that subcutaneous hemorrhage at abdominal acupoints in the acupuncture group resolved spontaneously without treatment. The patients in the control group had transient diarrhea, which was relieved spontaneously on the second day without affecting the continuation of treatment.

## Discussion

AP is an acute abdominal condition caused by damage to the pancreas, which exerts digestion-promoting effects on glands and surrounding organs, and it is mainly characterized by a local inflammatory reaction in the pancreas, which may lead to organ dysfunction. In developed countries, obstruction of the common bile duct caused by gallstones and alcoholism is the commonest cause of AP (40). In China, cholelithiasis is the main cause of the development of AP, followed by high levels of blood triglycerides and excessive consumption of alcohol. The typical symptoms of AP are acute and persistent severe upper abdominal pain accompanied by abdominal distension, nausea, and vomiting. A laboratory examination shows elevated levels of serum amylase and lipase, and typical early manifestations on imaging include pancreatic edema and/or peripancreatic tissue necrosis (41, 42).

Acupuncture has a history of thousands of years in China and is an important part of TCM. In recent decades, as a complementary and alternative medicinal therapy acupuncture has been used worldwide to induce analgesia and improve the functioning of the gastrointestinal tract (9, 43). A Acupuncture can not only treat physical pain (44), but can also play an important role in the treatment of visceral hyperalgesia (45). Clinically, acupuncture has been recognized to be an effective way to treat abdominal pain and distension in irritable bowel syndrome (IBS) (46). Studies have shown that electroacupuncture can relieve visceral hypersensitivity caused by stress in rats with IBS and can produce analgesic effects (47). Moreover, the neural mechanism of the effect of acupuncture on gastrointestinal motility mainly involves nerve fiber conduction and the sympathetic, parasympathetic, enteric, and central nervous systems (43).

It has been reported that electroacupuncture can reduce stress-induced delays in gastric emptying and can inhibit stress-induced acceleration of colonic transport (48). Acupuncture at the Siguan acupoints (LI4 and LR3) can not only reverse increases in gastrointestinal motility induced by mosapride citrate (49), but can also enhance gastrointestinal motility after inhibition by loperamide (50). It can be seen that acupuncture has a double regulatory effect that depends on the intensity of gastrointestinal motility. Moreover, acupuncture can also regulate gastrointestinal function *via* its effects on the gastrointestinal barrier (51, 52), visceral hypersensitivity (53, 54), and the brain–gut axis (55, 56).

Gastrointestinal dysfunction such as abdominal pain and distension is the main clinical manifestation of AP. The induction of analgesia is the focus of the treatment of AP. Opioids and non-steroidal drugs are commonly used as analgesic and anti-inflammatory agents, but evidence of the efficacy and safety of various analgesic drugs in the treatment of AP is limited. At present, there are few guidelines and little consensus on the treatment of AP with analgesics (57). In recent years, clinical studies and animal experiments have shown that acupuncture has significant anti-inflammatory and analgesic effects on AP. Some researchers have pointed out that electroacupuncture at the Zusanli (ST36) and Zhigou (TE6) acupoints can substantially reduce the severity of abdominal pain and distension in patients with SAP and paralytic ileus and has high safety. This method can be recommended for the treatment of SAP in combination with western medicine (58). Electroacupuncture at abdominal acupoints is superior to that at limb acupoints in relieving abdominal pain (59). With regard to anti-inflammatory effects, Xue et al. (60) suggested that acupuncture at the ST25 acupoint might have therapeutic effects in rats with SAP by inhibiting the expression of nuclear factor-κB and reducing the release of pro-inflammatory cytokines. Zhang et al. (61) suggested that electroacupuncture alleviates inflammatory responses in AP by activating the cholinergic anti-inflammatory pathway based on the vagus nerve.

### Selection of Meridians and Acupoints

A total of 30 different acupoints were used in 19 RCTS, mainly involving the Stomach Meridian of Foot-Yangming (ST), the Large Intestine Meridian of Hand-Yangming (LI), And Ren Meridian (RN). The top 5 acupoints were Zusanli (ST36), Zhongwan (RN12), Tianshu (ST25), Hegu (LI4), and Neiguan (PC6). AP lesions in the abdomen, abdominal pain and abdominal distension belong to gastrointestinal dysfunction. Thus, the main meridians selected for acupuncture were ST, LI, and RN. ST36 and ST25 are important acupoints in Stomach Meridian, which mainly treat gastrointestinal diseases. RN12 belongs to Ren Meridian, located in the abdomen, and is good at treating digestive system diseases, such as abdominal pain, abdominal distention, vomiting and constipation. LI4 is the yuan-primary point of the Large Intestine Meridian, and is usually used in combination with ST36 to increase efficacy. PC6 is one of the commonly used acupoints on the pericardium meridian of the Hand-Jueyin. It is used in conjunction with ST36 and RN12 to treat stomachache, vomiting and diarrhea. Therefore, the final selection of meridians and acupoints was based on disease differentiation and treatment. Acupuncture treatment of AP with abdominal pain abdominal distension was an objective combination of modern science and acupuncture and moxibustion theory.

### Discussion of Main Results

Nineteen RCTs were included in this SR and MA. The results showed that, in comparison with the control treatment, acupuncture in combination with RT increased the total effectiveness rate, reduced the VAS scores for abdominal pain and distension, and reduced the time until relief of abdominal pain and distension. However, in comparison with RT, moxibustion had no benefit in terms of reducing the duration of abdominal pain in patients with AP. In addition, we found that acupuncture in combination with RT substantially reduced the time until recovery of bowel sound, time until first defecation, and length of hospital stay. Subgroup analysis showed that acupuncture substantially reduced the APACHE II score after treatment for 7–14 days. Of all the studies, only five mentioned adverse events. No additional adverse events were found to have been caused by acupuncture, except subcutaneous bleeding.

In the assessment of the risk of bias in the 19 RCTs, randomization was reported in more than half of the studies (57.89%), but the details of the randomization procedure were not specified. Only four studies (21.05%) reported allocation concealment. Double blinding is difficult to achieve in acupuncture. Sham or placebo acupuncture may be a good choice for blinding, even if it may have a placebo effect to some extent. Evidence shows that the therapeutic effect of acupuncture is persistent. Although other factors besides the specific effects of needling at the correct acupoints play an important role in the treatment effect, decreases in pain following acupuncture cannot be explained solely in terms of placebo effects (62).

We conducted subgroup analysis and sensitivity analysis for results with high heterogeneity, which may have affected the robustness of the results because of inconsistencies in acupuncture prescriptions and doctors' experience and habits. However, some researchers have pointed out that variations in the effect size between different trials of acupuncture are mainly driven by differences in the treatments received by the control group rather than differences in the characteristics of acupuncture treatment (62). Despite the low levels of evidence in this SR and MA, greater efficacy was observed when patients with AP and symptoms of abdominal pain and distension were treated by acupuncture in combination with RT, and few adverse events were observed. The commonest adverse events caused by acupuncture were pain and bruising, which disappeared spontaneously several days after acupuncture was stopped (63). Therefore, we still recommend acupuncture for the treatment of AP.

### Strengths and Limitations

To the best of our knowledge, this is the first SR and MA of the efficacy and safety of acupuncture for alleviating abdominal pain and distension in patients with AP. This study was systematically evaluated in strict accordance with the PRISMA report checklist. We did a comprehensive search of literature sources and provided all retrieval strategies. Heterogeneity tests and extensive subgroup analyses were fully performed, and differences in treatment duration and interventions may be important influencing factors. The risk of bias and quality of evidence were described in detail. Thus, the results of our study may be more reliable than those of a small-scale study. However, there are still some limitations. Firstly, the quality of the original studies was low, and the heterogeneity in the results was high. Heterogeneity was still present after subgroup analysis, and it was difficult to find the source of the heterogeneity. Secondly, no clinical trial registration number was mentioned in any of the original studies, and the needle, acupoints, manipulations, and course of treatment that were selected in each study could not be combined. Therefore, heterogeneity in the acupuncture protocol may have led to the statistical heterogeneity in the samples. Thirdly, we searched as many databases as possible, but we could not find unpublished studies with negative results. Funnel plots and Egger's-test showed that publication bias was present. We tried to perform a sensitivity analysis of the results using the trim-and-fill method. Because there are many reasons for publication bias, which may affect our judgment of the results, further studies are needed to verify our conclusions and conduct more specific analyses.

## Conclusion

In conclusion, the results of the current SR and MA suggest that, in comparison with RT, acupuncture has significant efficacy and safety when used for alleviating abdominal pain and distension in patients with AP. In order to improve the quality of RCTs, it is possible to generalize the results of acupuncture studies more effectively by summarizing effective acupoints or stimulation points on the body surface and formulating a standardized operation plan.

## Data Availability Statement

The original contributions presented in the study are included in the article/Supplementary Material, further inquiries can be directed to the corresponding author.

## Author Contributions

FZ and XZ: study conception and design. LL and DC: administrative support. XZ, ZL, YZ, HY, DG, LY, and XW: collection and assembly of data. FZ and SY: data analysis, interpretation, and manuscript writing. All authors: final approval of manuscript.

## Funding

This study was funded by the Key Discipline Construction Project of Sichuan Administration of Traditional Chinese Medicine (No. 202072).

## Conflict of Interest

The authors declare that the research was conducted in the absence of any commercial or financial relationships that could be construed as a potential conflict of interest.

## Publisher's Note

All claims expressed in this article are solely those of the authors and do not necessarily represent those of their affiliated organizations, or those of the publisher, the editors and the reviewers. Any product that may be evaluated in this article, or claim that may be made by its manufacturer, is not guaranteed or endorsed by the publisher.
