# Supplementary material for: Acupuncture for Relieving Abdominal Pain and Distension in Acute Pancreatitis: A Systematic Review and Meta-Analysis
Source: Front Psychiatry. 2021 Dec 3;12:786401. doi: 10.3389/fpsyt.2021.786401 (PMC8678533; doi:10.3389/fpsyt.2021.786401)
Supplement: Supplementary file 1 [file Data_Sheet_1.pdf]

Sensitivity analysis and Egger’s test

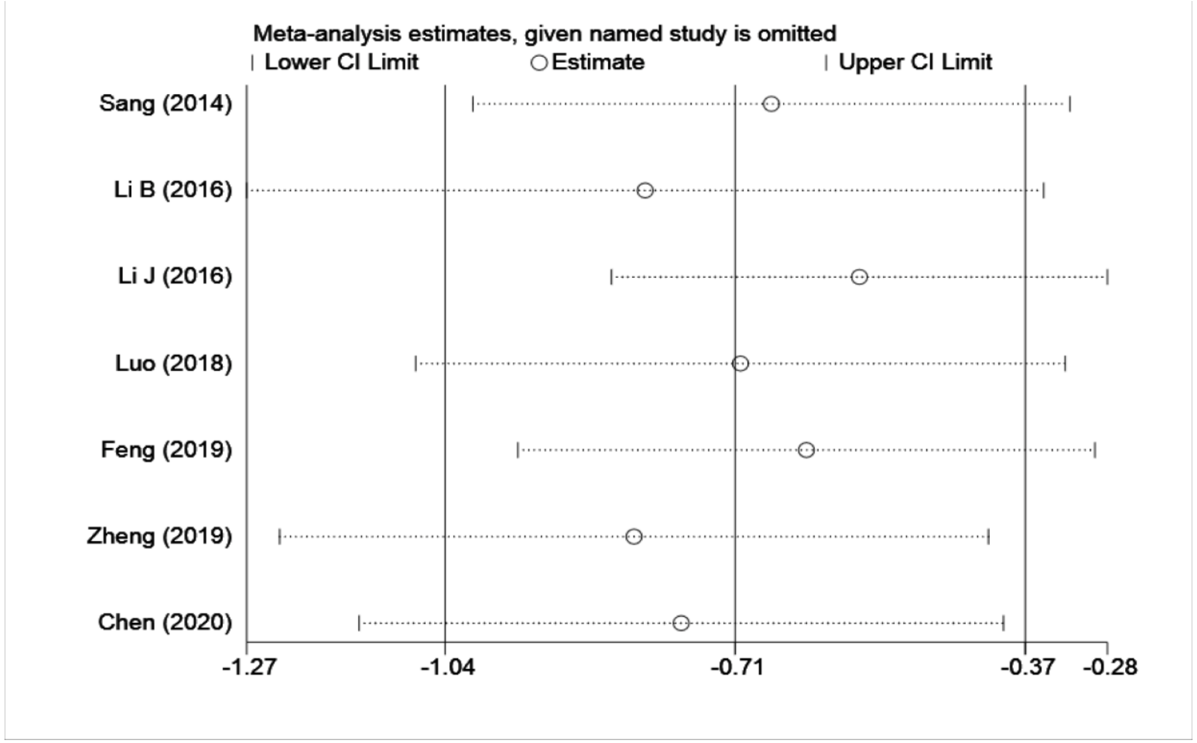


Sensitivity analysis of VAS score for abdominal distension.

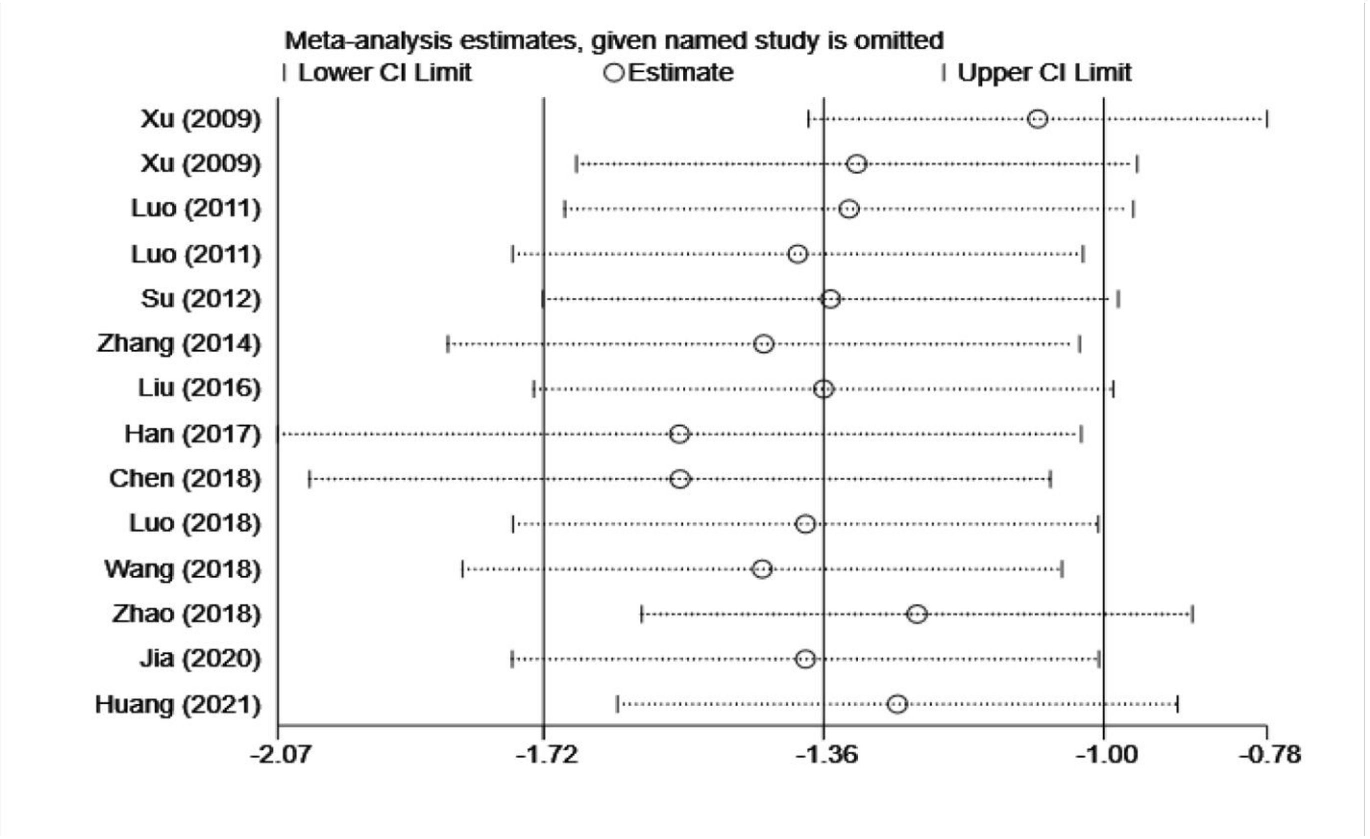


Sensitivity analysis of time until relief of abdominal pain.

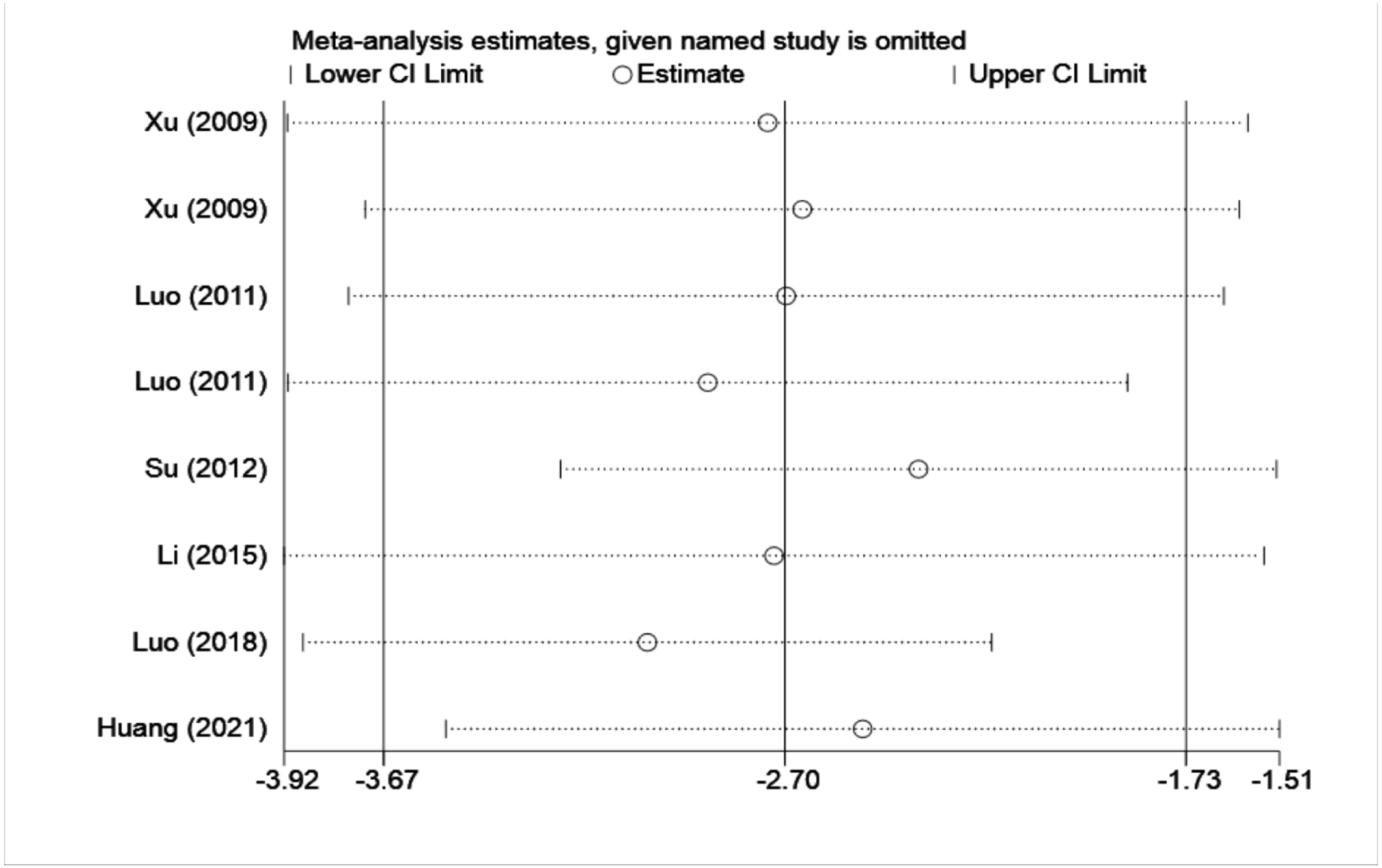


Sensitivity analysis of time until relief of abdominal distension.

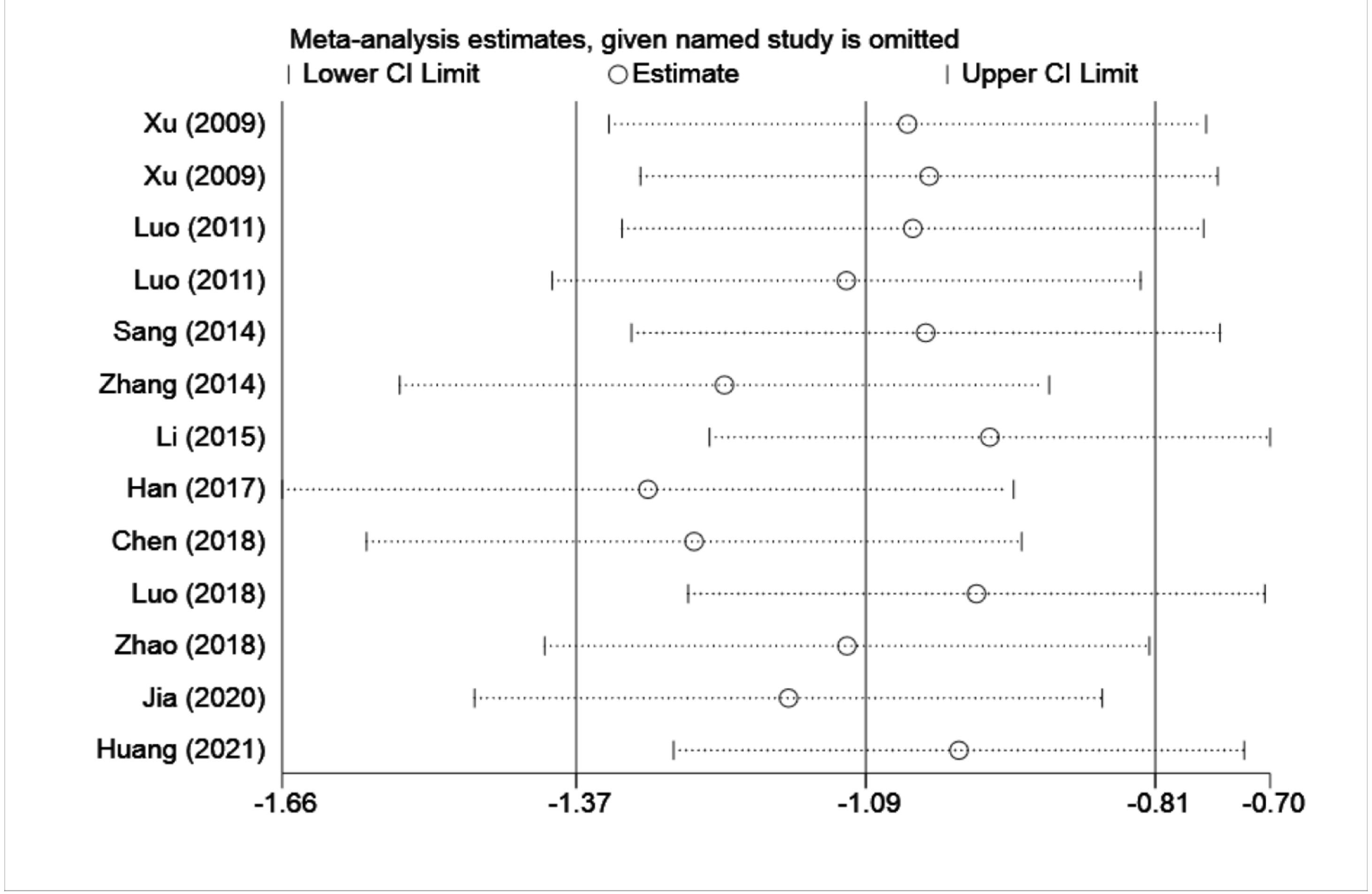


Sensitivity analysis of time until recovery of bowel sound.

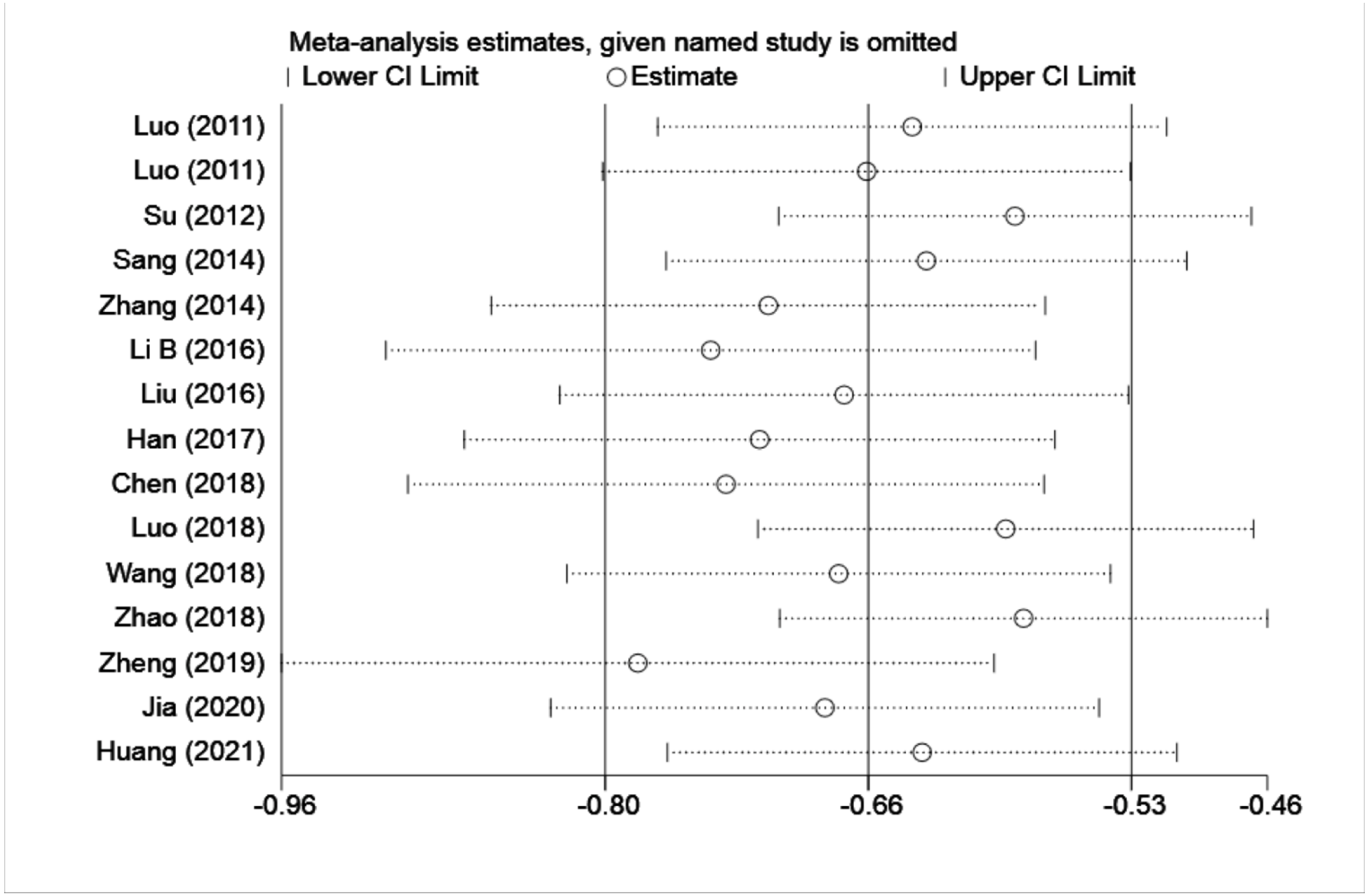


Sensitivity analysis of time until first defecation.

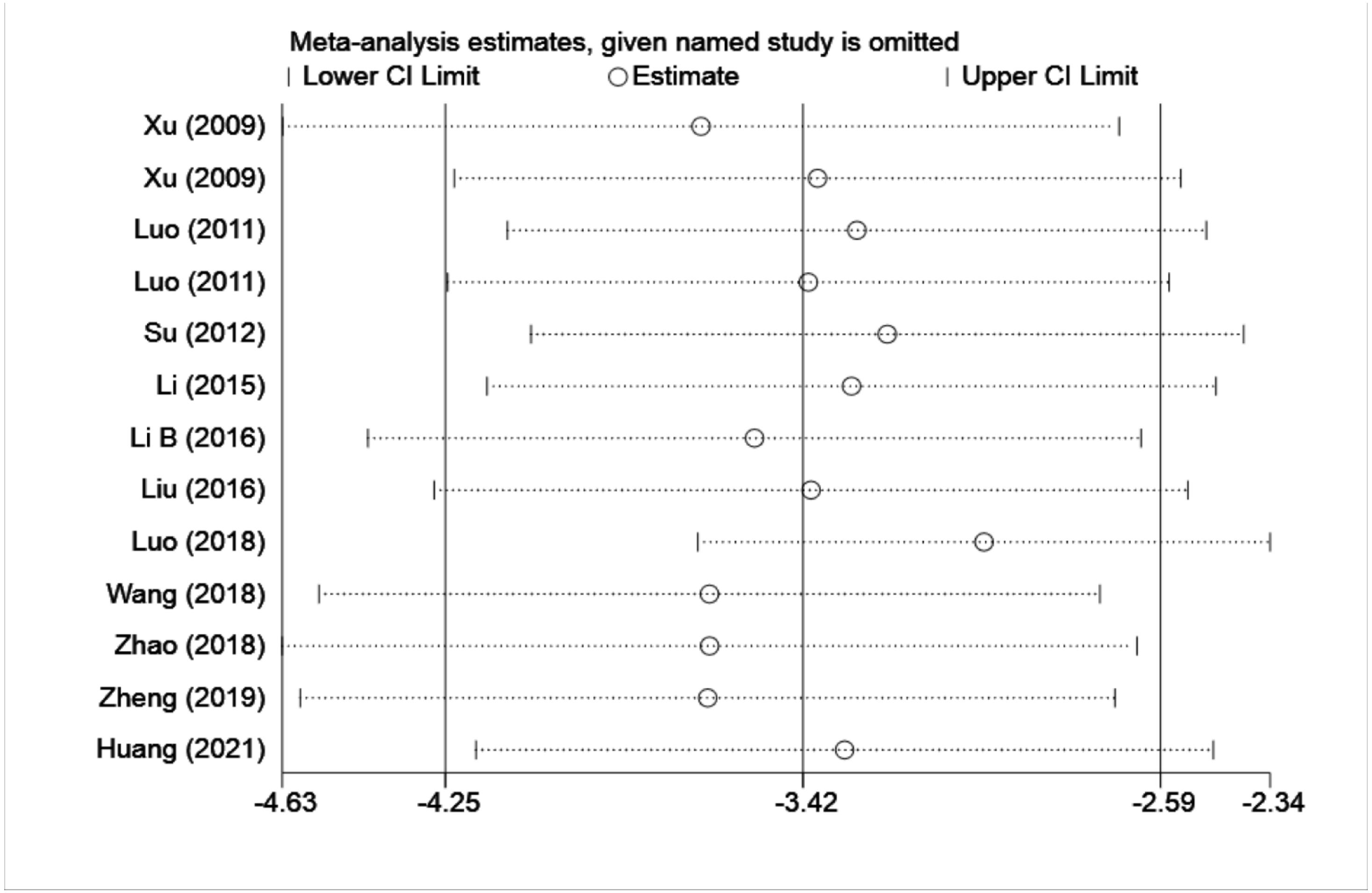


Sensitivity analysis of length of hospital stay.

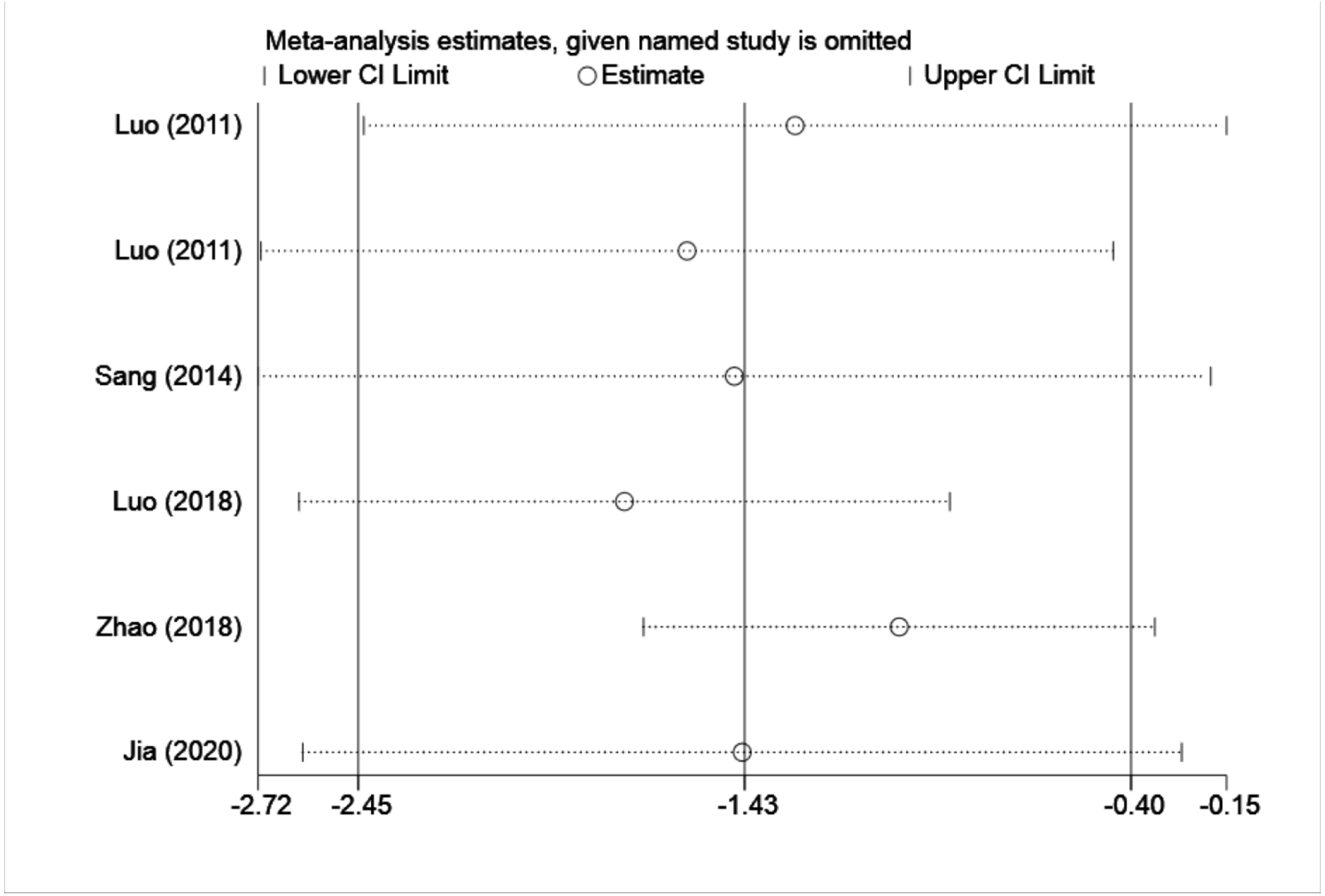


Sensitivity analysis of APACHE II score.

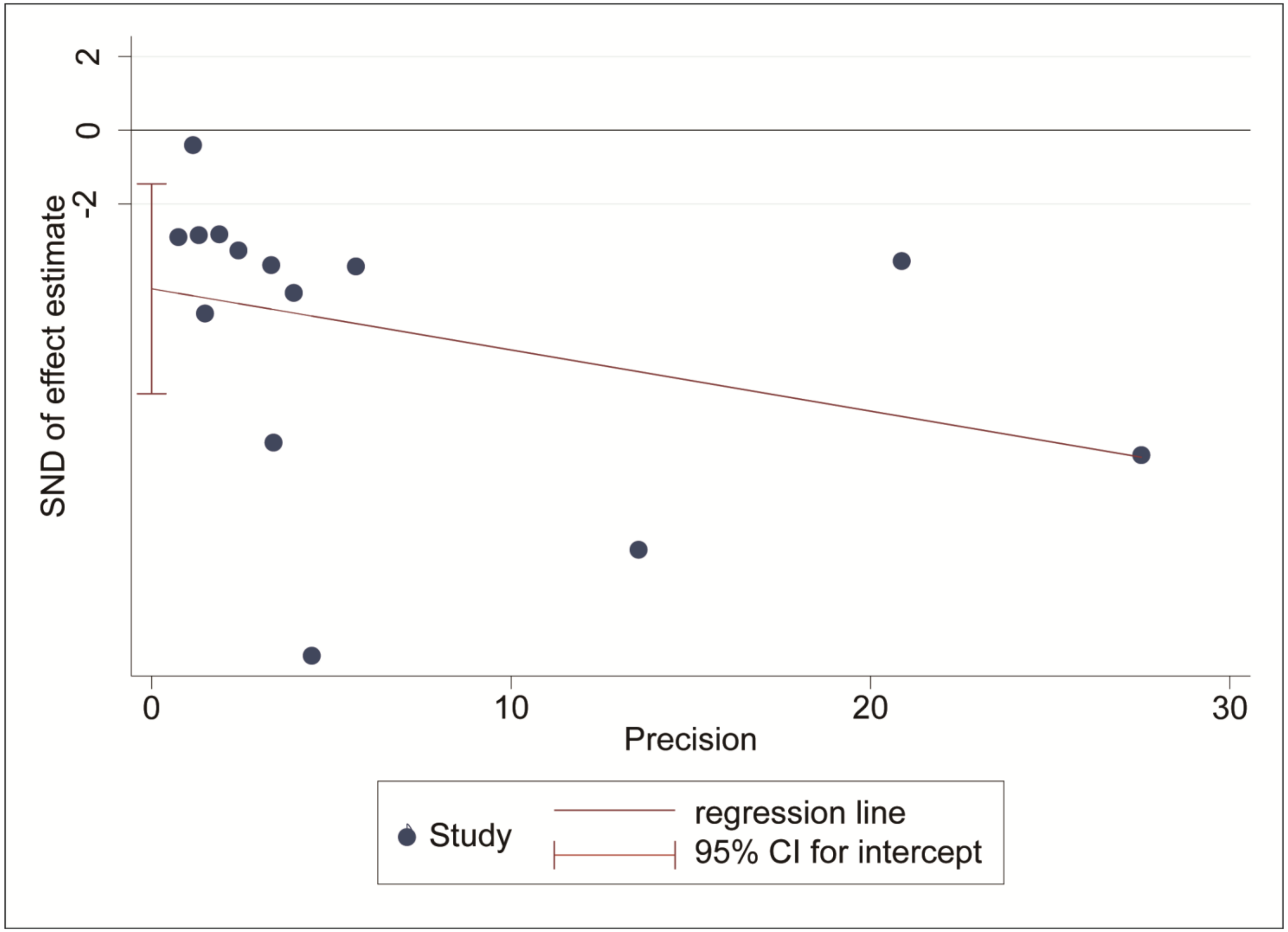


Egger's test of the time until relief of abdominal pain.
