# Supplementary material for: Acupuncture for Relieving Abdominal Pain and Distension in Acute Pancreatitis: A Systematic Review and Meta-Analysis
Source: Front Psychiatry. 2021 Dec 3;12:786401. doi: 10.3389/fpsyt.2021.786401 (PMC8678533; doi:10.3389/fpsyt.2021.786401)
Supplement: Supplementary file 2 [file Data_Sheet_2.PDF]

PubMed Search strategy:

Search number	Query
#1	Acute pancreatitis[MeSH Terms]
#2	Acute pancreatitis[Text Word]
#3	#1 OR #2
#4	Abdominal pain[MeSH Terms]
#5	Abdominal distension[MeSH Terms]
#6	#4 AND #5
#7	#4 OR #5 OR #6
#8	Acupuncture[MeSH Terms]
#9	Acupuncture therapy[MeSH Terms]
#10	Acupuncture points[MeSH Terms]
#11	electro puncture[MeSH Terms]
#12	Moxibustion[MeSH Terms]
#13	(Acupressure[Text Word]) OR (moxibustion[Text Word])
#14	ear acupuncture[MeSH Terms]
#15	warm acupuncture[MeSH Terms]
#16	#8 OR #9 OR #10 OR #11 OR #12 OR #13 OR #14 OR #15
#17	trial[All fields]
#18	randomized[All fields]
#19	controlled clinical trial[All fields]

#20	randomized controlled trial[All fields]
#21	#17 OR #18 OR #19 OR #20
#22	#3 AND #7 AND #16 AND #21

The Cochrane Library Search strategy:

Search number	Query
#1	(Acute pancreatitis):ti,ab,kw
#2	Abdominal pain:ti,ab,kw
#3	Abdominal distension:ti,ab,kw
#4	(Abdominal pain):ti,ab,kw AND (Abdominal distension):ti,ab,kw
#5	#2 OR #3 OR #4
#6	Acupuncture:ti,ab,kw
#7	Acupuncture therapy:ti,ab,kw
#8	Acupuncture points:ti,ab,kw
#9	electro puncture:ti,ab,kw
#10	Moxibustion:ti,ab,kw
#11	Acupressure:ti,ab,kw
#12	ear acupuncture:ti,ab,kw

#13	warm acupuncture:ti,ab,kw
#14	#6 OR #7 OR #8 OR #9 OR #10 OR #11 OR #12 OR #13
#15	#1 AND #4 AND #14
#16	randomized controlled trial:ti,ab,kw
#17	controlled clinical trial:ti,ab,kw
#18	randomized:ti,ab,kw
#19	trial:ti,ab,kw
#20	#16 OR #17 OR #18 OR #19
#21	#15 AND #20

Web of science Search strategy:

Search number	Query
1	ALL=(Acute pancreatitis)
2	ALL=(Abdominal pain)
3	ALL=(Abdominal distension)
4	#2 AND #3
5	#2 OR #3 OR #4

6	ALL=(Acupuncture)
7	ALL=(Acupuncture therapy)
8	ALL=(Acupuncture points)
9	ALL=(electro puncture)
10	ALL=(Moxibustion)
11	ALL=(Acupressure)
12	ALL=(ear acupuncture)
13	ALL=(warm acupuncture)
14	#6 OR #7 OR #8 OR #9 OR #10 OR #11 OR #12 OR #13
15	ALL=(randomized controlled trial)
16	ALL=(controlled clinical trial)
17	ALL=(randomized)
18	ALL=(trial)
19	#15 OR #16 OR #17 OR #18
20	#1 AND #5 AND #14 AND #19

Embase database Search strategy:

Search number	Query
#1	Acute pancreatitis OR Acute pancreatitis
#2	Abdominal pain OR Abdominal distension
#3	Acupuncture OR Acupuncture therapy OR Acupuncture points OR electro puncture OR Moxibustion OR Acupressure OR moxibustion OR ear acupuncture OR warm acupuncture
#4	Randomized controlled trial OR controlled clinical trial OR randomized or trial
#5	#1 AND #2 AND #3 AND #4

SinoMed database Search strategy:

"急性胰腺炎"[全部字段:智能] AND ("腹痛"[全部字段:智能] OR "腹胀"[全部字段:智能]) AND ("针刺"[全部字段:智能] OR "针灸"[全部字段:智能] OR "艾灸"[全部字段:智能] OR "电针"[全部字段:智能] OR "耳针"[全部字段:智能] OR "温针灸"[全部字段:智能]) AND ("临床研究"[全部字段:智能] OR "临床观察"[全部字段:智能] OR "疗效"[全部字段:智能])

CNKI database Search strategy:

(SU%'急性胰腺炎') and (SU%'腹痛'+'腹胀') and (SU%'针刺'+'针灸'+'艾灸'+'电针'+'耳针'+'温针灸') and (SU%'临床研究'+'临床观察'+'疗效')

Wanfang database Search strategy:

主题=(急性胰腺炎) AND 主题=(腹痛 OR 腹胀) AND 主题=(针刺 OR 针灸 OR 艾灸 OR 电针 OR 耳针 OR 温针灸) AND 主题=(临床研究 OR 临床观察 OR 疗效)

VIP database Search strategy:

U=(急性胰腺炎) AND U=(腹痛 OR 腹胀) AND U=(针刺 OR 针灸 OR 艾灸 OR 电针 OR 耳针 OR 温针灸) AND U=(临床研究 OR 临床观察 OR 疗效)
