# Supplementary material for: Acupuncture for Relieving Abdominal Pain and Distension in Acute Pancreatitis: A Systematic Review and Meta-Analysis
Source: Front Psychiatry. 2021 Dec 3;12:786401. doi: 10.3389/fpsyt.2021.786401 (PMC8678533; doi:10.3389/fpsyt.2021.786401)
Supplement: Supplementary file 3 [file Data_Sheet_3.PDF]

Exclusion list

Citation	Reason for exclusion
Zhu Wei-fang. Effect of electroacupuncture on long-term rehabilitation of patients with acute pancreatitis [J].Modern Digestion & Intervention, 2019,(S2):2601-2602.	The index is not satisfied
She Chang-hua, Chen Qian-yun, Deng Xiu-juan. Effects of abdominal acupuncture and moxibustion combined with rhubarb nasal feeding on recovery of intestinal function of moderate to severe acute pancreatitis with intestinal paralysis [J]. HANSHAO JIBING ZAZHI, 2021,28(2):73-75.	The index is not satisfied
Zhao Yue-meng. Clinical randomized controlled study of distal acupoint selection in the improvement of abdominal pain and abdominal distention in acute pancreatitis [D]. Chengdu University of Chinese Medicine,2020.	Intervention is not satisfied
Li Sheng-fa, Liu Xi-rong, Zhou Heng, et al. Effect of acupuncture and moxibustion combined with Western medicine on acute pancreatitis [J]. Shanghai J Acup Moxib, 201,40(2):168-173.	Intervention is not satisfied
Yu Xiao-tang, QU Xiao-yu. Clinical observation and nursing care of acupoint moxibustion in the adjuvant treatment of acute pancreatitis [J]. China's Naturopathy,2014,22(2):10-11.	Intervention is not satisfied
Wang Lu-e, Ding Kai-fang, Yang Dong-xing, et al. Clinical observation of moxibustion combined with moxibustion in patients with acute pancreatitis with gastrointestinal dysfunction [J]. Gems of Health,2021,(11):8.	Unclear criteria
Huang Hong-lan, Zhou Ting-mei, Cao Yi, et al. Effect of electroacupuncture combined with Traditional Chinese and Western medicine on acute pancreatitis [J]. Gems of Health,2019,(23):128-129.	Unclear criteria
Xue You-ping, Jiang Li, Huang Teng-hui, et al. Clinical study on the diagnosis and treatment of acute pancreatitis at pancreatic point[J]. Chin Acup Moxib,2002,22(12):815-817.	Not RCT
Xue You-ping, Jiang Li, Huang Ten-hui, et al. Clinical study of pancreatic point in the treatment of acute pancreatitis [J]. J Gansu Coll Tradit Chin Med,2004,21(4):25-27.	Content repetition
